# *Plasmodium falciparum* infection induces T cell tolerance that is associated with decreased disease severity upon re-infection

**DOI:** 10.1084/jem.20241667

**Published:** 2025-04-11

**Authors:** Diana Muñoz Sandoval, Florian A. Bach, Alasdair Ivens, Adam C. Harding, Natasha L. Smith, Michalina Mazurczyk, Yrene Themistocleous, Nick J. Edwards, Sarah E. Silk, Jordan R. Barrett, Graeme J.M. Cowan, Giorgio Napolitani, Nicholas J. Savill, Simon J. Draper, Angela M. Minassian, Wiebke Nahrendorf, Philip J. Spence

**Affiliations:** 1 Institute of Immunology and Infection Research, University of Edinburgh, Edinburgh, UK; 2 Instituto de Microbiologia, Universidad San Francisco de Quito, Quito, Ecuador; 3 Weatherall Institute of Molecular Medicine, University of Oxford, Oxford, UK; 4 The Jenner Institute, University of Oxford, Oxford, UK; 5 Department of Biochemistry and Kavli Institute for Nanoscience Discovery, University of Oxford, Oxford, UK; 6 NIHR Oxford Biomedical Research Centre, Oxford, UK

## Abstract

Immunity to severe malaria is acquired quickly, operates independently of pathogen load, and represents a highly effective form of disease tolerance. The mechanism that underpins tolerance remains unknown. We used a human rechallenge model of *falciparum* malaria in which healthy adult volunteers were infected three times over a 12 mo period to track the development of disease tolerance in real-time. We found that parasitemia triggered a hardwired innate immune response that led to systemic inflammation, pyrexia, and hallmark symptoms of clinical malaria across the first three infections of life. In contrast, a single infection was sufficient to reprogram T cell activation and reduce the number and diversity of effector cells upon rechallenge. Crucially, this did not silence stem-like memory cells but instead prevented the generation of cytotoxic effectors associated with autoinflammatory disease. Tolerized hosts were thus able to prevent collateral tissue damage in the absence of antiparasite immunity.

## Teaser

Sandoval et al. show that a single malaria episode reprograms T cell activation to minimize cytotoxicity and injury during subsequent infections. This adaptation is long-lived, operates independently of pathogen load, and associates with immunity to severe forms of the disease.

## Introduction

The epidemiology of human malaria clearly shows that immunity develops in two distinct phases. First, individuals acquire protection against severe life-threatening disease, and in areas of high transmission this occurs very quickly (often before 12 mo of age) (Gonçalves et al., 2014; Moxon et al., 2020; Snow and Marsh, 1998; Snow et al., 1998). Then after many years of exposure protection against clinical malaria is established, which promotes the transition to asymptomatic infection (usually in adolescence) (Doolan et al., 2009). This temporal dissociation between clinical immunity and immunity to severe malaria suggests that they are underpinned by different mechanisms of host defense. In agreement, clinical immunity often coincides with control of parasitemia (and can therefore be supported by mechanisms of host resistance) whereas immunity to severe malaria is acquired independently of pathogen load and is a form of disease tolerance (Gonçalves et al., 2014). One leading hypothesis suggests that broadly neutralizing antibodies that recognize variant surface antigens associated with severe malaria (such as EPCR-binding PfEMP1) could prevent severe disease without affecting total pathogen load (Moxon et al., 2020; Reyes et al., 2024). In this scenario, immunity to severe malaria would depend upon the rapid production of antibodies that can specifically eliminate pathogenic variants. At present, there is limited in vivo evidence that such broad cross-reactivity can be achieved or that neutralizing antibodies can be produced within the first year of life to inhibit cytoadherence and reduce sequestration (McLean et al., 2024; Tuju et al., 2019). The mechanism that underpins disease tolerance in human malaria therefore remains unknown.

An alternative explanation is that the host response to infection is quickly modified to minimize the harm caused by malaria parasites. It is well known that metabolic adaptations are induced to increase host fitness during the blood cycle (Cumnock et al., 2018; Ramos et al., 2019; Wang et al., 2018), and control of inflammation might provide an additional path toward disease tolerance (Medzhitov et al., 2012). In support of this idea, inflammation decreases with exposure in children (Ademolue et al., 2017), and mice can modify their immune response to repeated infection to minimize tissue stress and toxicity independently of pathogen load (Nahrendorf et al., 2021). Nevertheless, there is currently no evidence that host adaptations can persist after pathogen clearance to provide long-term protection in people.

Controlled human malaria infection (CHMI) (Stanisic et al., 2018) offers a unique opportunity to investigate mechanisms of disease tolerance. Healthy malaria-naive volunteers are inoculated with live parasites and their response is tracked throughout infection by repeated sampling; volunteers are inoculated with the same clonal parasite line (with known variant surface antigen expression) to remove parasite genotype/phenotype as confounding variables; and infections can be terminated at the same parasitemia to maintain a consistent pathogen load. Importantly, this threshold can be safely set at 5,000–10,000 parasites ml^−1^ to challenge the immune system with >5 × 10^7^ parasites (Milne et al., 2021). We, therefore, developed a human rechallenge model of malaria to track the development of disease tolerance in real-time and hypothesized that volunteers would learn to control inflammation and minimize pathology to compensate for their lack of pathogen control.

## Results

### The risk of severe malaria decreases exponentially with exposure

**Figure S1.**
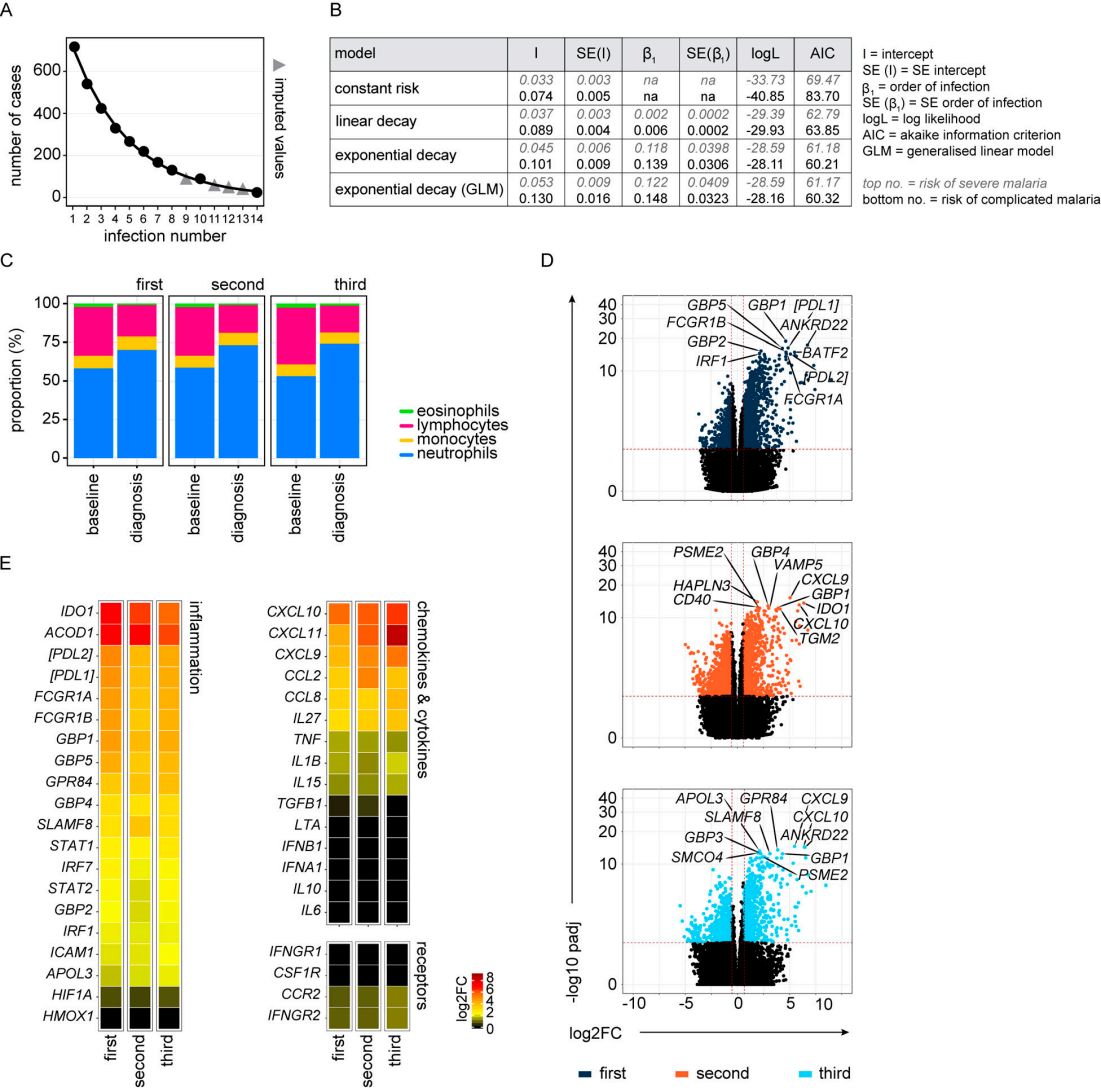
**Systemic inflammation is not attenuated upon rechallenge. (A)** Data were extracted from Gonçalves et al. (2014) to examine the frequency of severe or complicated malaria during the first 14 infections of life in infants living in a hyperendemic setting. We first plotted the total number of cases of malaria (including mild or uncomplicated episodes) and used least squares regression to impute missing values (for infection numbers 9, 11, 12, and 13). Imputed values are shown as a grey triangle whereas filled circles indicate data as reported by Gonçalves et al. The total number of children experiencing at least one episode of malaria was *n* = 715. **(B)** We next plotted the incidence of severe or complicated malaria at each order of infection (as shown in Fig. 1, A and B) and performed maximum likelihood estimation to select the best model fit for these data; log likelihood (logL) and AIC both show that an exponential decay in risk provides the best fit (SE, standard error). **(C)** Healthy malaria-naive adults were enrolled in the VAC063 study and infected up to three times with *P. falciparum* (clone 3D7) by direct blood challenge. The mean frequency of eosinophils, lymphocytes, monocytes, and neutrophils in whole blood at baseline and diagnosis is shown (note that the loss of lymphocytes at diagnosis is comparable in all three infections). **(D)** RNAseq was used to identify differentially expressed genes in whole blood at diagnosis (versus baseline) (adj P < 0.05 and >1.5 fold-change). Volcano plots show all differentially expressed genes (colored dots) and the dashed lines represent the significance/fold-change (FC) cutoffs. The top 10 differentially expressed genes (lowest adj P) in each infection are labeled (first, second, and third infection were analyzed independently). **(E)** The log_2_ fold-change of signature genes associated with interferon signaling and type I inflammation are shown at diagnosis (versus baseline) in the first, second, and third infection. Square brackets indicate that common protein names have been used. In C, *n* = 10 (first and second infection) and *n* = 6 (third infection). In D and E, *n* = 10 (first infection), *n* = 9 (second infection), and *n* = 6 (third infection). v1040 was excluded from RNAseq analysis in the second infection because their baseline sample failed QC.

**Figure 1.**
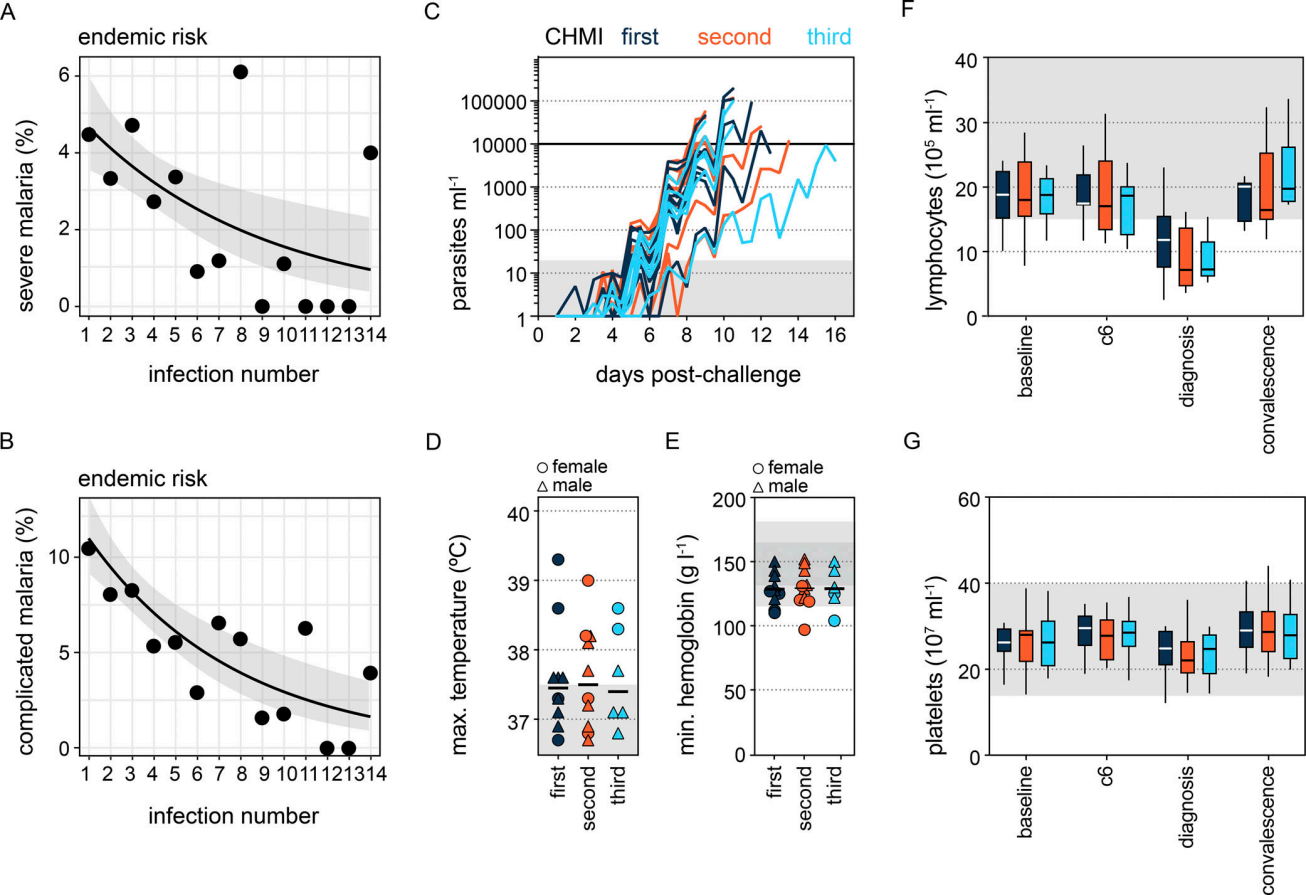
**The risk of severe malaria decreases exponentially with exposure. (A and B)** Data were extracted from Gonçalves et al. (2014) to examine the frequency of severe (A) or complicated (B) malaria during the first 14 infections of life in infants living in a hyperendemic setting. We performed maximum likelihood estimation to select the best model fit for these data; the black line shows the best fit and grey shading represents the 95% confidence intervals. In both cases, an exponential decay provided a better fit than either a linear decay or constant risk. In A, *n* = 102 severe episodes and in B, *n* = 199 complicated episodes of malaria (see Fig. S1, A and B for case imputation and model performance). **(C–G)** Healthy malaria-naive adults were infected up to three times with *P. falciparum* (clone 3D7) by direct blood challenge. Repeated sampling before, during, and after each infection allowed us to track the development of disease tolerance in real-time. **(C)** Parasite growth curves for first, second, and third infection; each line represents a volunteer, and lines are color-coded by infection number. Parasite density was measured in peripheral blood by qPCR every 12 h. The grey box represents the lower limit of quantification (20 parasites ml^−1^) and the treatment threshold of 10,000 parasites ml^−1^ is denoted by the black line. **(D and E) **Maximum core body temperature (D) and minimum hemoglobin (E) were recorded during each infection (up to 48 h after treatment). Each symbol represents one volunteer with a line shown at the median. Grey shading indicates a normal range (115–165 g liter^−1^ hemoglobin for females and 130–180 g liter^−1^ hemoglobin for male volunteers). **(F and G)** Lymphocytes (F) and platelets (G) were quantified in circulation the day before infection (baseline), 6 days after challenge (c6), at the peak of infection (diagnosis), and ∼1 mo after drug treatment (convalescence). Boxplots show the median and interquartile range (IQR), and whiskers represent the 95% confidence intervals. Sample size (*n*) is 10 for the first and second infections and *n* = 6 for the third infection. There was no statistically significant difference between groups using a significance threshold of 5% (Kruskal–Wallis test). These data are also presented in Salkeld et al. (2022).

To estimate the number of times we would need to infect volunteers to observe host adaptations in a CHMI study, we first asked how quickly mechanisms of disease tolerance could be established in an area of endemicity. We reanalyzed data from a prospective cohort study undertaken in an area of high transmission in Tanzania (Gonçalves et al., 2014). Here, the authors performed longitudinal sampling with active case detection in >800 infants and recorded each independent infection (including pathogen load and disease severity) from birth to 4 years of age. Crucially, there was no reduction in pathogen load across the study period as measured by microscopy (circulating parasitemia) or HRP2 ELISA (total parasite biomass). Nonetheless, the authors described a rapid decrease in the incidence of severe malaria consistent with acquired immunity. To ask how quickly the risk of severe malaria decreased, we performed maximum likelihood estimation to select the best model fit for these data (Fig. S1, A and B). We asked whether the risk of severe malaria remained constant, decreased linearly or exponentially with exposure, or decreased suddenly after *n* infections (where *n* = 1, 2, 3, etc.). The latter stepwise model performed poorly and was removed from further analysis. In contrast, the linear and exponential models provided a good fit and performed better than a constant risk model. To increase our sample size, we repeated this analysis and included cases of moderately severe malaria—we collectively describe these episodes of malaria as complicated as 87.7% led to hospitalization. Once again, the linear and exponential models provided a good fit with the latter performing better by both log-likelihood and Akaike information criterion (AIC). Taken together, our results demonstrate that the risk of severe or complicated malaria is highest during the first infection of life and decreases exponentially thereafter (Fig. 1, A and B). Host adaptations that promote disease tolerance should therefore be apparent after a single malaria episode.

### Developing a human rechallenge model of malaria

With this in mind, we infected volunteers two or three times in our CHMI study and used the most virulent human parasite *Plasmodium falciparum*. 10 healthy malaria-naive adults were recruited and infected by intravenous injection of parasitized red blood cells during the VAC063A and VAC063B clinical trials; all returned for rechallenge later between 4 and 8 mo (during the VAC063B and VAC063C trials, respectively). Six volunteers who took part in both VAC063A and VAC063B were infected for a third time during VAC063C. A blood challenge model was chosen because it standardizes the infectious dose, prolongs the period of blood-stage infection (cf. mosquito challenge), and removes liver-stage immunity as a possible confounding factor (Draper et al., 2018; Duncan and Draper, 2012). Importantly, we used a recently mosquito-transmitted parasite line (<3 blood cycles from liver egress) (Cheng et al., 1997) since mosquitoes have been shown to reset *Plasmodium* virulence (Spence et al., 2013; Wichers-Misterek et al., 2023). Volunteers attended clinic the day before infection (baseline), every 12 h from the day after infection until diagnosis (the peak of infection), and then during the period of drug treatment, which was initiated within 12 h of diagnosis. These frequent visits allowed for regular blood sampling to construct a detailed longitudinal time course of each infection. Importantly, we found that the parasite multiplication rate (and peak parasitemia) were comparable between the first, second, and third malaria episodes (Fig. 1 C and Table S1). Furthermore, we found no significant change in symptoms or the frequency or severity of pyrexia, anemia, lymphopenia, or thrombocytopenia (Fig. 1, D–G). These data thus show that healthy adults do not acquire mechanisms of resistance (to reduce their pathogen load) and remain susceptible to clinical malaria. Our homologous rechallenge model therefore recapitulates the key features of endemic malaria in early life.

### Infection triggers a hardwired innate immune response

**Figure 2.**
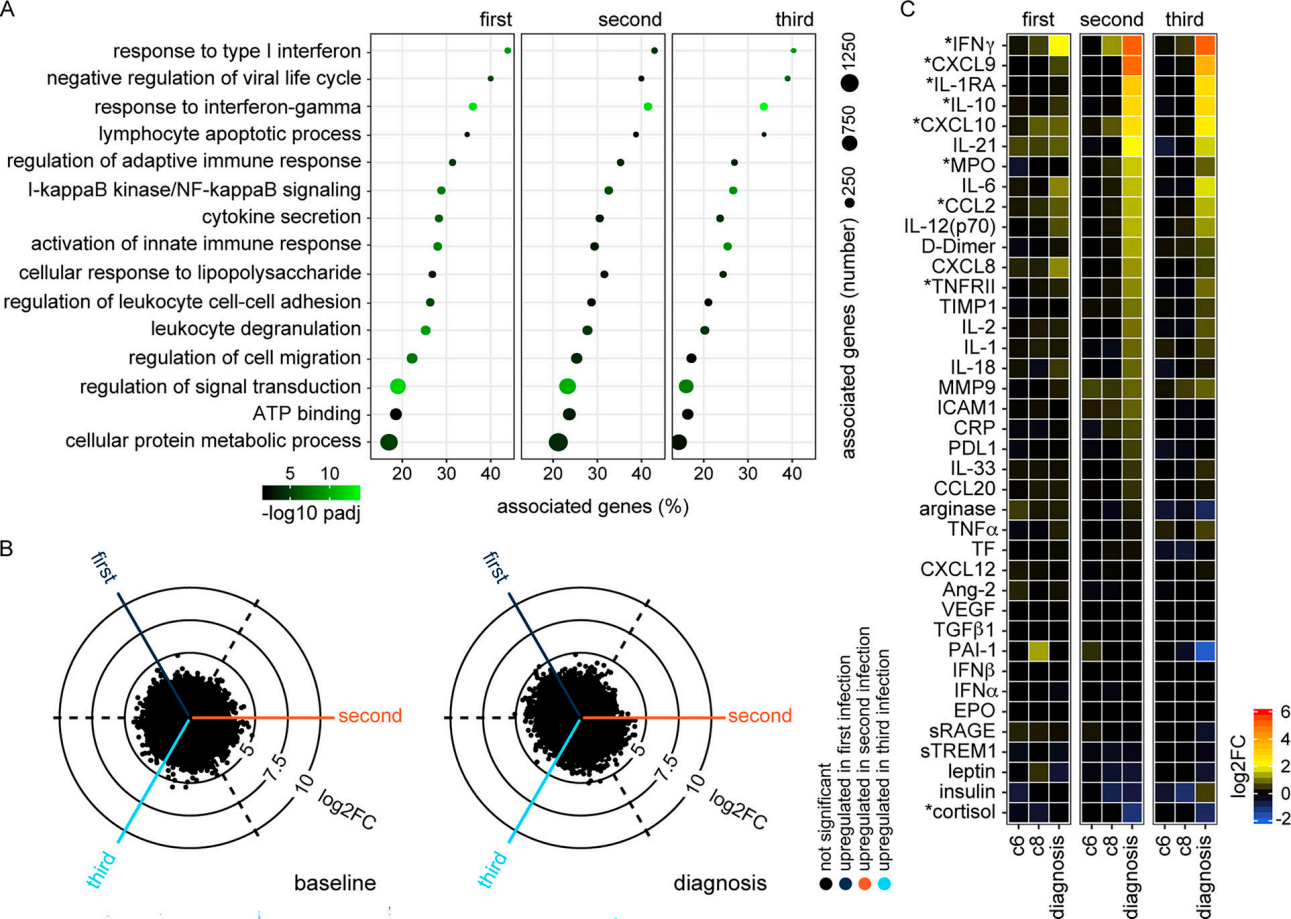
**Infection triggers a hardwired innate immune response. (A)** RNAseq was used to identify differentially expressed genes in whole blood at diagnosis (versus baseline) (adj P < 0.05 and >1.5 fold-change). ClueGO was then used for functional gene enrichment analysis and placed significant GO terms into functional groups by relatedness. Shown are the leading GO terms from 15 non-redundant groups with the lowest adj P value in the first infection. The same GO terms are plotted in the second and third infections (note that each infection was analyzed independently). **(B)** Radar plots (or three-way volcano plots) show the number of differentially expressed genes in whole blood between each infection—the left plot compares all baseline samples and the right plot diagnosis. Dashed lines represent the center point for each volcano plot, and the position of each dot relative to this line shows up- or downregulation. There were no differentially expressed genes in any of the six pairwise comparisons (adj P < 0.05 and >1.5 fold-change [FC]). **(C)** 39 plasma analytes were quantified before and during each infection using a highly multiplexed bead-based assay. The log_2_ fold-change of each analyte is shown relative to baseline on day 6 and 8 after challenge (c6 and c8, respectively) and at diagnosis. Analytes are ordered by log_2_ fold-change and are marked with an asterisk if they varied significantly during both the second and third (compared to first) infections (adj P < 0.05 by linear regression with Benjamini–Hochberg correction for multiple testing). In A and B, *n* = 10 (first infection), 9 (second infection), and 6 (third infection). v1040 was excluded from RNAseq analysis in the second infection because their baseline sample failed QC. In C, *n* = 9 (first and second infection) and 5 (third infection). v1040 was excluded from plasma analysis because all samples failed QC.

The absence of antiparasite immunity means that any change in the host response to infection cannot be attributed to a reduced number of circulating parasites. Our model therefore provides the ideal setting in which to investigate mechanisms of disease tolerance. One potential route to tolerance might be to reduce or restrict systemic inflammation, and we, therefore, used whole blood RNA-sequencing (RNAseq) and DESeq2 (Love et al., 2014) to identify differentially expressed genes during the first, second, and third infection. We found a remarkably similar pattern of interferon-stimulated gene expression regardless of infection number (Fig. S1, C–E). Furthermore, functional gene enrichment analysis showed that the hierarchy of Gene Ontology (GO) terms was near-identical (Fig. 2 A). These transcriptional signatures were consistent with the rapid recruitment of activated monocytes and neutrophils into peripheral blood, which has been extensively described in naive hosts infected with *P. falciparum* (Feintuch et al., 2016; Loughland et al., 2020) and *Plasmodium vivax* (Antonelli et al., 2014; Rocha et al., 2015), but it was surprising to see no obvious change upon rechallenge. Nevertheless, by analyzing each infection independently, it was possible that we were missing important quantitative differences, so we performed direct pairwise comparisons between the first, second, and third infections. Initially, we compared each preinfection time point to identify season-dependent shifts in baseline gene expression, and we found zero differentially expressed genes between infections (adj P < 0.05 and absolute fold-change >1.5). When we then compared each diagnosis time point to identify adaptations in the host response, we again found zero differentially expressed genes (Fig. 2 B); evidently, the first three infections of life trigger a hardwired innate response that is not influenced by season or previous exposure.

Nonetheless, host control of inflammation may not be transcriptionally regulated, so we directly measured systemic inflammation at protein level using a highly multiplexed custom bead assay (39 plasma analytes indicative of inflammation, coagulation, oxidative stress, and metabolism). By analyzing the concentration of each analyte through time, we could fit mixed-effects models to test the hypothesis that inflammation was attenuated upon rechallenge. What we actually found, however, was that many of the prototypical products of monocyte and neutrophil activation (such as CXCL10, IL-1RA, and MPO) were increased in the second and third infections (Fig. 2 C). The same was true for hallmark cytokines associated with innate lymphoid cell or T cell activation (such as IFNγ). Collectively, these data demonstrate that *P. falciparum* triggers a hardwired innate immune response throughout the first three infections of life. And crucially, we find no evidence that systemic inflammation is attenuated.

### A single infection attenuates T cell activation

We therefore moved on to examine adaptive T cells, which are inherently plastic, proliferative, and long-lived, and uniquely placed to quickly and permanently alter the host response to infection. The acute phase response to malaria causes extreme lymphopenia leading to a 30–70% loss of circulating cells at the peak of infection (Fig. 1 F). The majority of these are recruited to the inflamed spleen (Hviid et al., 1997; Mandala et al., 2022), so it is difficult to assess T cell activation and differentiation at diagnosis. Instead, we need to analyze T cell activation after drug treatment when the emergency response begins to resolve and T cells return to circulation (Bach et al., 2023). At this time point, analyzing T cell phenotypes in peripheral blood can provide a readout of tissue-specific immune responses. Post-treatment blood samples were not available from the VAC063A or VAC063B clinical trials but were collected during VAC063C. In this trial, we recruited new malaria-naive controls to provide time-matched samples from the first infection and infected 11 volunteers contemporaneously (three first infection, two second infection, and six third infection) (Table S1). As such, we switched to a cross-sectional analysis of VAC063C to incorporate posttreatment time points.

**Figure S2.**
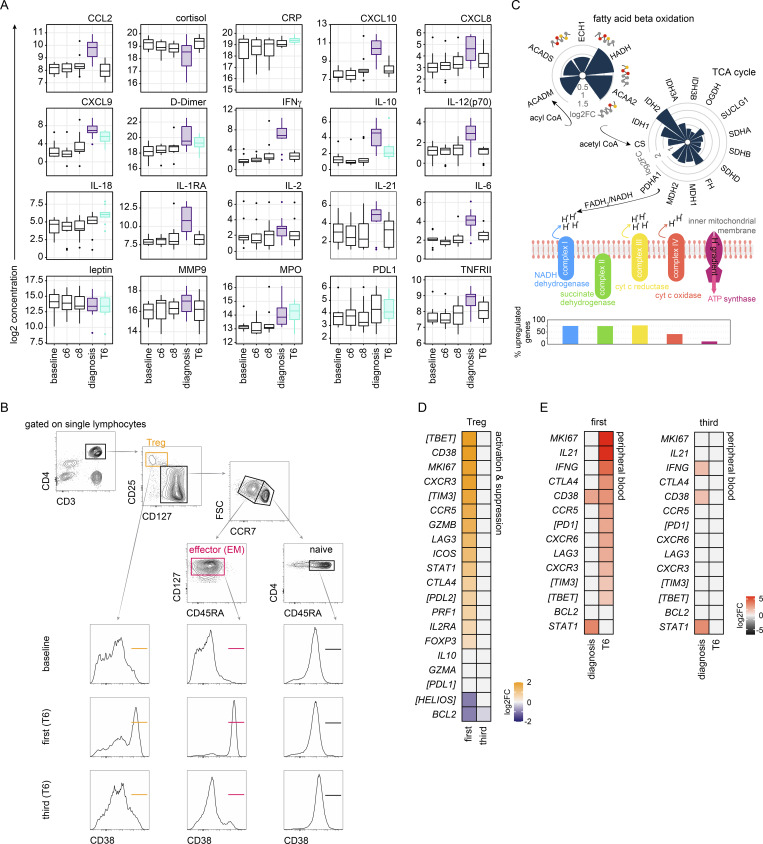
**Activated T cells are released into circulation as inflammation resolves. (A)** Mixed-effects models and linear regression were used to identify plasma analytes that vary significantly at diagnosis and/or T6 across the entire VAC063C dataset (all volunteers regardless of infection number). Kenward Roger approximation was used to calculate P values and multiple test correction was performed using the Benjamini–Hochberg method; significance (adj P < 0.05) is indicated by colored box plots (purple at diagnosis and turquoise at T6). Box (median and IQR) and whisker (1.5× upper or lower IQR) plots are shown with outliers as dots. **(B)** Gating strategy for sorting CD4^+^ T cell subsets during VAC063C. CD4^+^ T cells were sorted ex vivo (within 2 h of blood draw) into TRIzol for downstream RNAseq, and cells with a naive, effector (effector memory [EM]), or regulatory phenotype were sorted as shown at baseline and T6. Note that we did not use CD38 for sorting but subsequently used this marker to assess the level of activation within each subset at both time points. **(C)** RNAseq was used to analyze transcriptional regulation of fatty acid β-oxidation, the tricarboxylic acid (TCA) cycle, and oxidative phosphorylation (oxphos) in flow-sorted effector (effector memory) CD4^+^ T cells during first infection. The circular bar charts show the log_2_ fold-change (FC) of each major enzyme involved in fatty acid β-oxidation and the TCA cycle clockwise in reaction order 6 days after parasite clearance (T6 versus baseline). The vertical bar chart shows the proportion of oxphos enzymatic subunits that are transcriptionally upregulated at T6; all subunits required to form complex I to IV in the electron transport chain and ATP synthase are shown. The key molecules that connect these metabolic pathways are labeled. **(D)** RNAseq was used to identify differentially expressed genes in flow-sorted regulatory T cells (Treg) (CD4^pos^ CD25^hi^ CD127^neg^) in the first and third infection (T6 versus baseline) (adj P <0.05 and >1.5 fold-change). Heatmap shows the log_2_ fold-change of differentially expressed genes that control Treg activation and suppressor function. **(E)** RNAseq was used to identify differentially expressed genes in whole blood at diagnosis and T6 (relative to baseline) in the first and third infection (adj P < 0.05 and >1.5 fold-change). Signature genes associated with T cell activation, T_H_1 polarization, and cytokine production are shown. In A, *n* = 10 (3 first infection, 2 second infection, and 5 third infection). v1040 was excluded from plasma analysis because all samples failed QC. In C and D, *n* = 2 or 3 for the first infection (T6 and baseline, respectively) and *n* = 6 for the third infection (v313 was excluded at T6 because this sample failed QC). In E, *n* = 3 for the first infection and *n* = 6 for the third infection. In D and E, non-significant genes are displayed with a log_2_ fold-change of zero and square brackets indicate that common protein names have been used.

**Figure 3.**
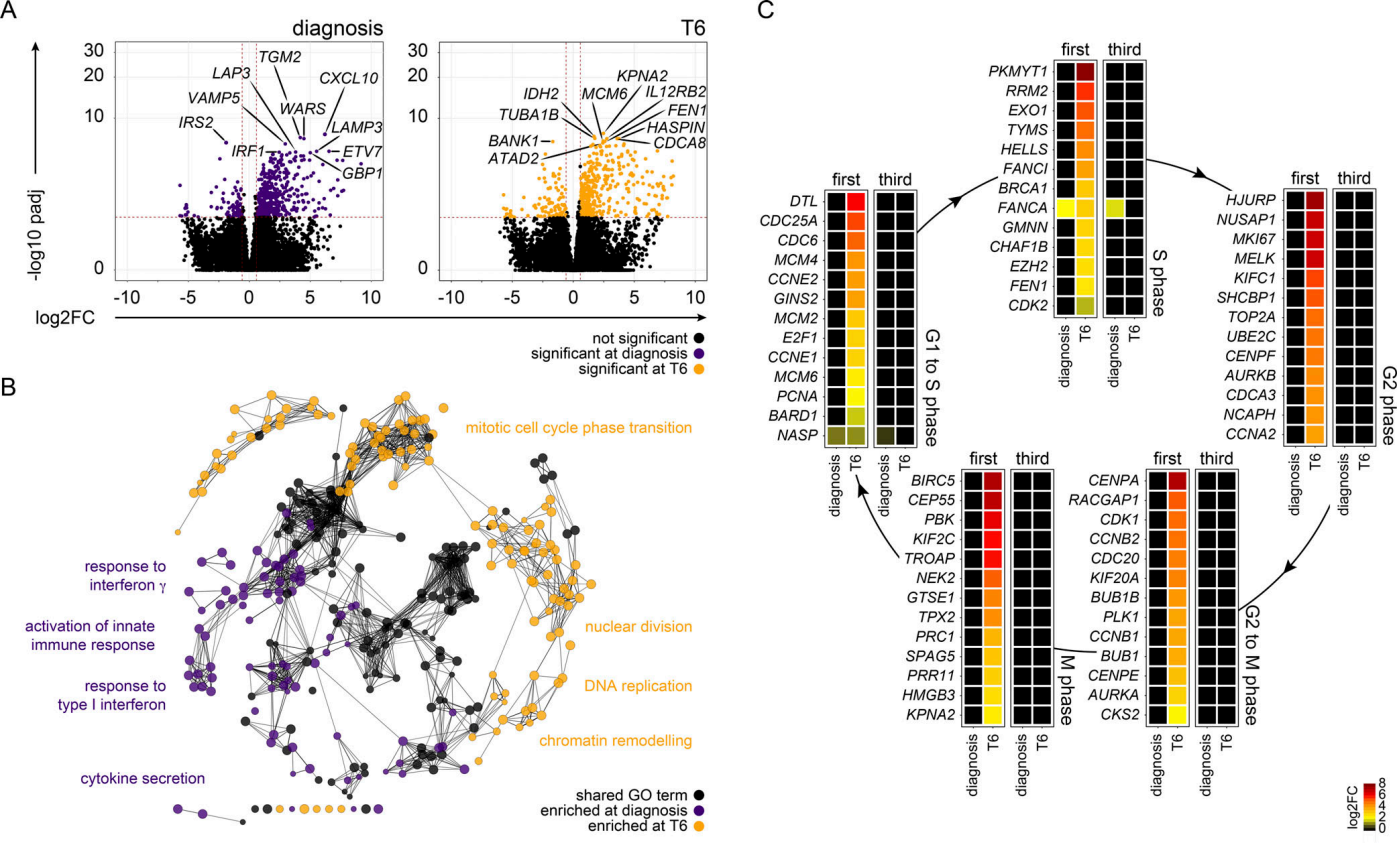
**Inflammation is followed by proliferation in the circulation. (A)** RNAseq was used to identify differentially expressed genes in whole blood at diagnosis and T6 (versus baseline) during first infection in the VAC063C study (adj P < 0.05 and >1.5 fold-change [FC]). Volcano plots show all differentially expressed genes (colored dots), and the dashed lines represent the significance/fold-change cutoffs. The top 10 differentially expressed genes (lowest adj P) at each time point are labeled. **(B)** Differentially expressed genes at T6 and diagnosis were combined for GO analysis of first infection and ClueGO was used to construct a merged functional gene ontology network. Each node represents a GO term and nodes are colored according to whether their associated genes were majoritively (>60%) derived from the diagnosis or T6 time point. GO terms that were shared between time points are shown in black. Four leading GO terms (each from a unique functional group) are labeled for each time point. **(C)** Heatmaps show differentially expressed genes at diagnosis and T6 (versus baseline) during first and third infection (adj P < 0.05 and >1.5 fold-change). The log_2_ fold-change of key genes associated with each phase of the cell cycle is shown. Non-significant genes are displayed with a log_2_ fold-change of zero. In A and B, *n* = 3 (first infection only), and in C, *n* = 3 (first infection) and *n* = 6 (third infection).

6 days after drug treatment (designated T6), lymphopenia had completely resolved and all other clinical symptoms of malaria (including fever) had receded. Furthermore, most of the circulating markers of systemic inflammation had returned to baseline, and this was observed in every volunteer regardless of infection number (Fig. S2 A). Nevertheless, we found a large transcriptional signature in whole blood, which did not overlap with the innate response captured at diagnosis. Instead, the differentially expressed genes at T6 had unique functional enrichment terms relating to cell cycle and nuclear division (Fig. 3, A and B). Remarkably, this proliferative burst was only observed in volunteers undergoing their first infection of life (Fig. 3 C).

**Figure 4.**
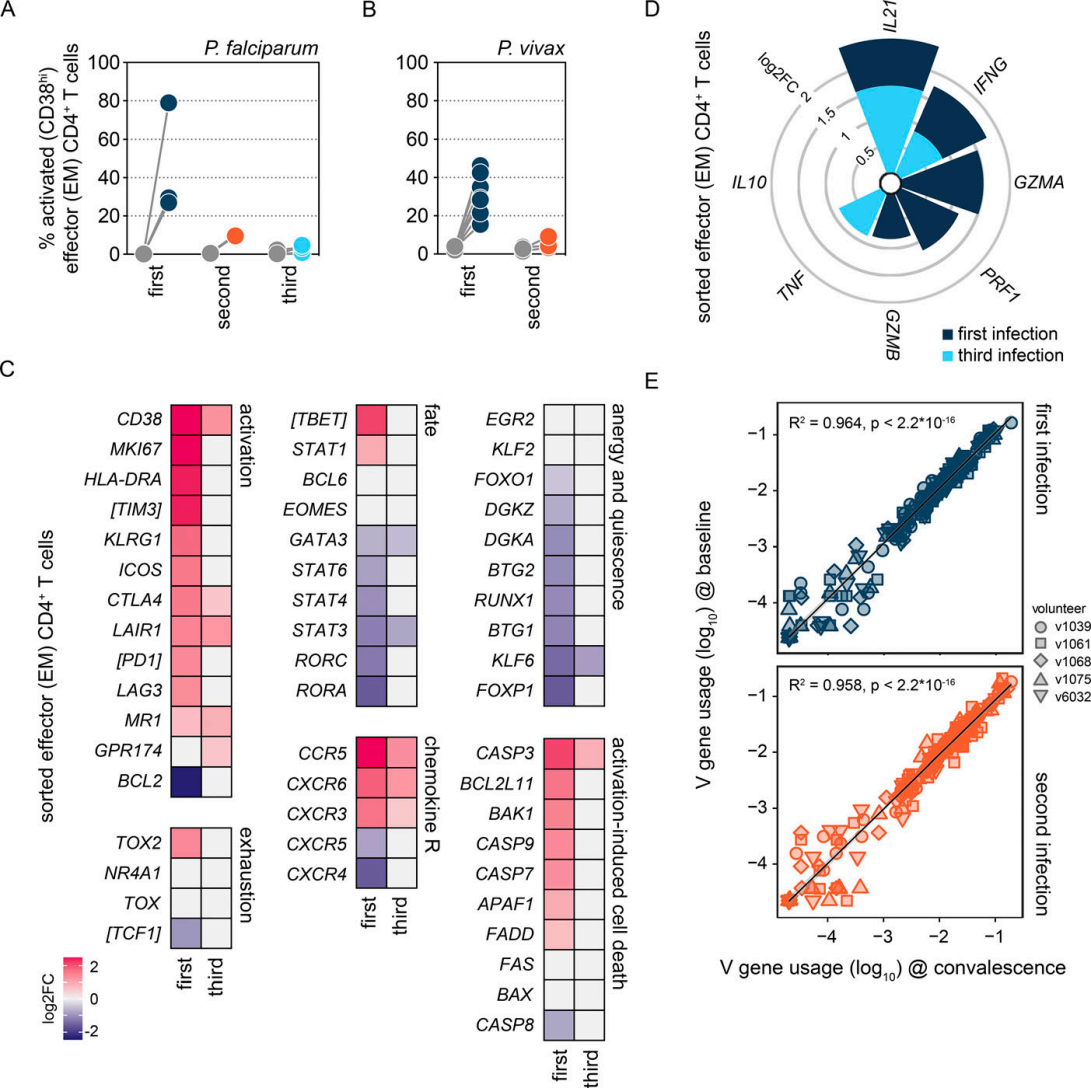
**A single infection attenuates T cell activation. (A)** The percentage of activated CD38^hi^ effector or effector memory (EM) CD4^+^ T cells was analyzed by flow cytometry at baseline (grey dots) and 6 days after parasite clearance (colored dots) during the VAC063C study. Data are shown for volunteers undergoing their first, second, or third infection of life (see Fig. S2 B for gating strategies). **(B)** The percentage of activated CD38^hi^ effector or effector memory CD4^+^ T cells at baseline (grey dots) and 6 days after parasite clearance (colored dots) during the VAC069 study. **(C and D)** RNAseq was used to identify differentially expressed genes in flow-sorted effector (effector memory) CD4^+^ T cells (T6 versus baseline) in first and third infection during VAC063C (adj P < 0.05 and >1.5 fold-change [FC]). The heatmaps in C show the log_2_ fold-change of markers of T cell activation and exhaustion and the master transcription factors that shape T cell fate. Non-significant genes are displayed with a log_2_ fold-change of zero and square brackets indicate that common protein names have been used. The stacked circular bar chart in D shows the log_2_ fold-change of cytokines and cytotoxic effector molecules. **(E)** Correlation between log10 transformed TRBV gene frequency at baseline and 28 days after challenge (convalescence) showing linear regression (black line) with 99% confidence intervals (grey shaded area). These data represent V gene usage across the entire T cell compartment and were obtained after one or two malaria episodes from volunteers who were subsequently infected for a third time during VAC063C. In A, *n* = 3 for the first infection, *n* = 2 for the second infection, and *n* = 6 for the third infection. In B, *n* = 8 for the first infection and *n* = 3 for the second infection. In C and D, *n* = 2 or 3 for the first infection (T6 and baseline, respectively) and *n* = 6 for the third infection (v313 was excluded at T6 because this sample failed QC). In E, *n* = 5 in the first and second infection (v1065 convalescence samples failed QC).

Myeloid cells are generally terminally differentiated and do not proliferate after their release from the bone marrow; it therefore seemed likely that our whole blood RNAseq data captured the return of activated T cells to circulation. To explore this further, we sorted CD4^+^ T cells with an effector or effector memory phenotype (CCR7^neg^ CD45RA^neg^), which has been shown to dominate the adaptive response to malaria in a naive host (Bach et al., 2023). Sorting was performed on whole blood 1 day before challenge and 6 days after drug treatment (Fig. S2 B). Analysis of the cell surface markers used for sorting revealed dramatic activation of CD4^+^ T cells in the first infection but not the second or third infections (Fig. 4 A). This finding was reproduced in a separate CHMI study (VAC069) (Bach et al., 2023; Minassian et al., 2021b) that infected volunteers with *P. vivax*, a different species of human malaria parasite that is evolutionarily divergent from *P. falciparum* (Sharp et al., 2020) (Fig. 4 B). Our data therefore reveal that the unique feature of a first-in-life malaria episode is fulminant CD4^+^ T cell activation and that this response is attenuated after a single infection.

### A single infection attenuates T_H_1 polarization

To explore the transcriptional landscape of activated CD4^+^ T cells, we undertook RNAseq on the sorted effector (effector memory) CD4^+^ T cells obtained from volunteers infected with *P. falciparum*. We found almost 6,000 differentially expressed genes at T6 in the first infection (adj P < 0.05 and >1.5 fold-change), and functional gene enrichment analysis showed that these cells were proliferative and had increased their capacity for oxidative phosphorylation (Fig. 4 C and Fig. S2 C). Furthermore, they had upregulated each of the major costimulatory and inhibitory receptors required to control their fate and increased their expression of the signature chemokine receptors and transcription factors associated with T helper 1 (T_H_1) polarization. What’s more, the cytokines IFNγ and IL-21 were both strongly induced, which could indicate that infection stimulates double producers (Carpio et al., 2020) or that follicular helper T (T_FH_) cells were also released from the spleen (Fig. 4 D).

In the third infection, IFNγ and IL-21 were once again significantly upregulated at T6 together with T_H_1-associated chemokine receptors (Fig. 4, C and D). Yet, the transcription factors that drive T_H_1 differentiation (T-bet and STAT1) were no longer induced, and neither were the costimulatory molecules or inhibitory receptors observed in first infection. We therefore asked what modifies CD4^+^ T cell activation to avert T_H_1 polarization upon rechallenge. We found no transcriptional evidence that they were quiescent or anergic in third infection and the transcription factors that epigenetically enforce exhaustion were not induced (Fig. 4 C). Furthermore, there was no evidence of activation-induced cell death, and TCRβ sequencing revealed near-identical repertoires before and after infection, which suggests that attenuation was not caused by the clonal deletion of activated cells (Fig. 4 E). There was also no evidence that activated CD4^+^ T cells were diverted toward a regulatory fate, for example, hallmarks of T regulatory 1 (T_R_1) differentiation (such as Eomes and IL-10) were absent (Fig. 4, C and D).

An alternative explanation could be suppression by conventional regulatory CD4^+^ T cells but our data showed that regulatory T cells (Tregs) were activated in the first (not third) infection (Fig. S2 D). It, therefore, appears that malaria-experienced hosts can launch a specialized adaptive T cell program that maintains cytokine production without causing extensive activation, proliferation, or T_H_1 polarization. And we can identify this modified T cell response by transcriptional profiling of whole blood (Fig. S2 E), which means it will be possible to identify tolerized hosts in an endemic setting without the need for complex cell isolation protocols.

### Stem-like memory CD4^+^ T cells respond to rechallenge

**Figure S3.**
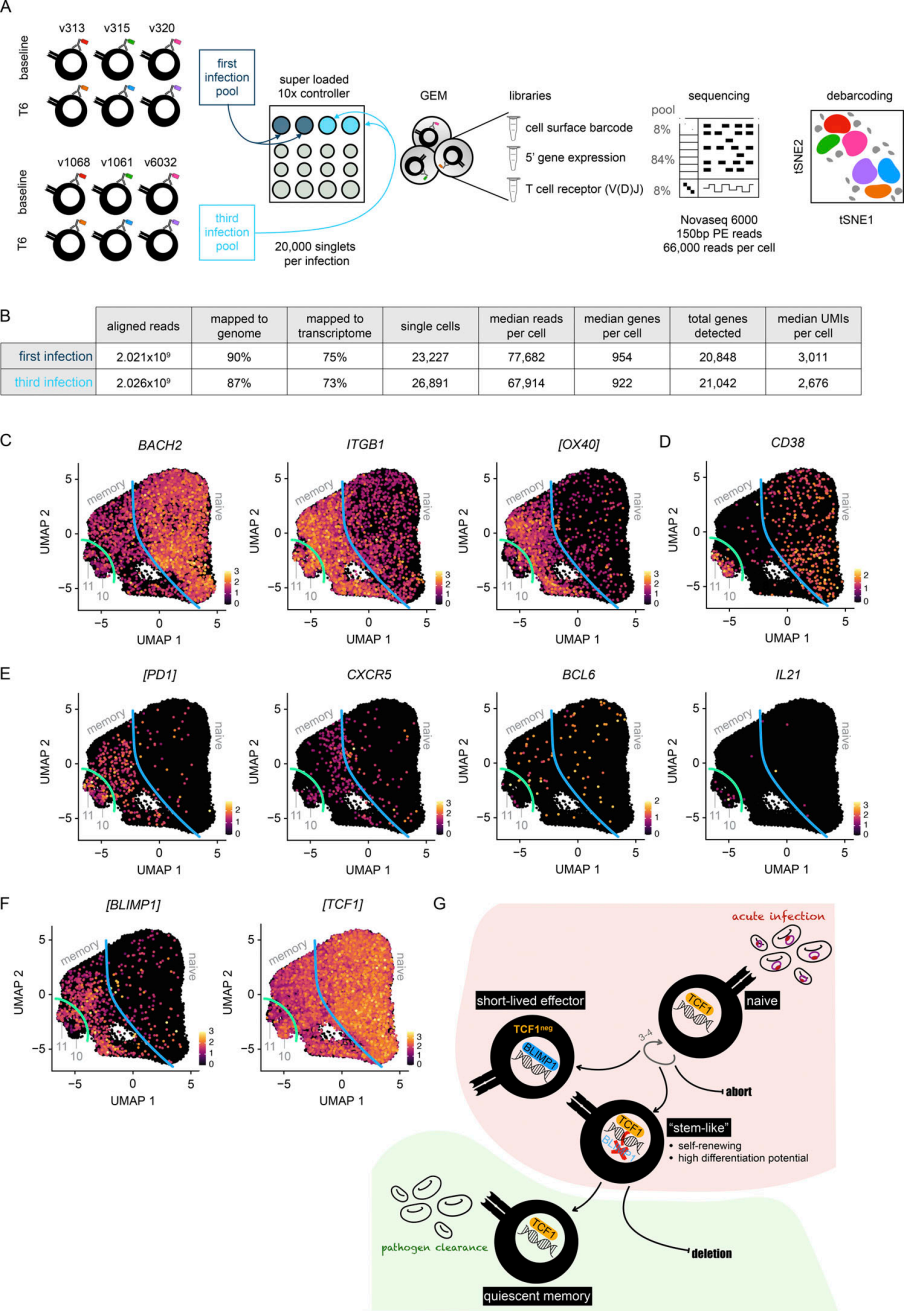
**Activated CD4** ^**+**^ **T cells bifurcate along the TCF1/BLIMP1 axis. (A)** VAC063C single-cell RNAseq workflow: each sample of flow-sorted CD4^+^ T cells was barcoded using TotalSeq-C oligo-tagged antibodies; samples from all volunteers and time points were pooled (separately for first and third infection); and pooled samples were superloaded onto a 10X Chromium Controller (we aimed to capture 30,000 singlets per pool). GEMs encapsulating a single cell (or doublets) were then generated and from each GEM three libraries were produced: (1) the cell surface barcode, (2) 5′ gene expression, and (3) TCR (after amplification of the V(D)J regions). Libraries were pooled at the specified ratios and sequenced. Finally, we used PCA-based clustering to debarcode all samples and remove doublets (see Materials and methods). **(B)** Cell Ranger was used to align 5′ gene expression and V(D)J sequencing reads (independently for the first and third infection). Shown is the output of Cell Ranger after removing doublets and performing QC. **(C–F)** Data from all volunteers and time points was concatenated for UMAP analysis. The expression intensity of markers for memory (C), activation (D), and follicular helper T (T_FH_) cell differentiation (E) are shown across the UMAP. The blue line represents the split between naive and memory cells whereas the green line represents the split between memory and activated cells. In F, the expression intensity of the master transcription factors associated with terminal differentiation (BLIMP1) versus the maintenance of stem-like properties (TCF1) are shown. In all cases, each UMAP is equivalent to those shown in Fig. 5 (for cross-reference) and square brackets indicate that common protein names have been used. **(G)** Proposed model of T cell activation during a first-in-life malaria episode. The maintenance of stem-like T cells is essential for long-lived memory; this requires sustained expression of TCF1 to repress BLIMP1 and prevent the terminal differentiation of short-lived effector cells. In A–F, *n* = 3 for first and third infection.

**Figure 5.**
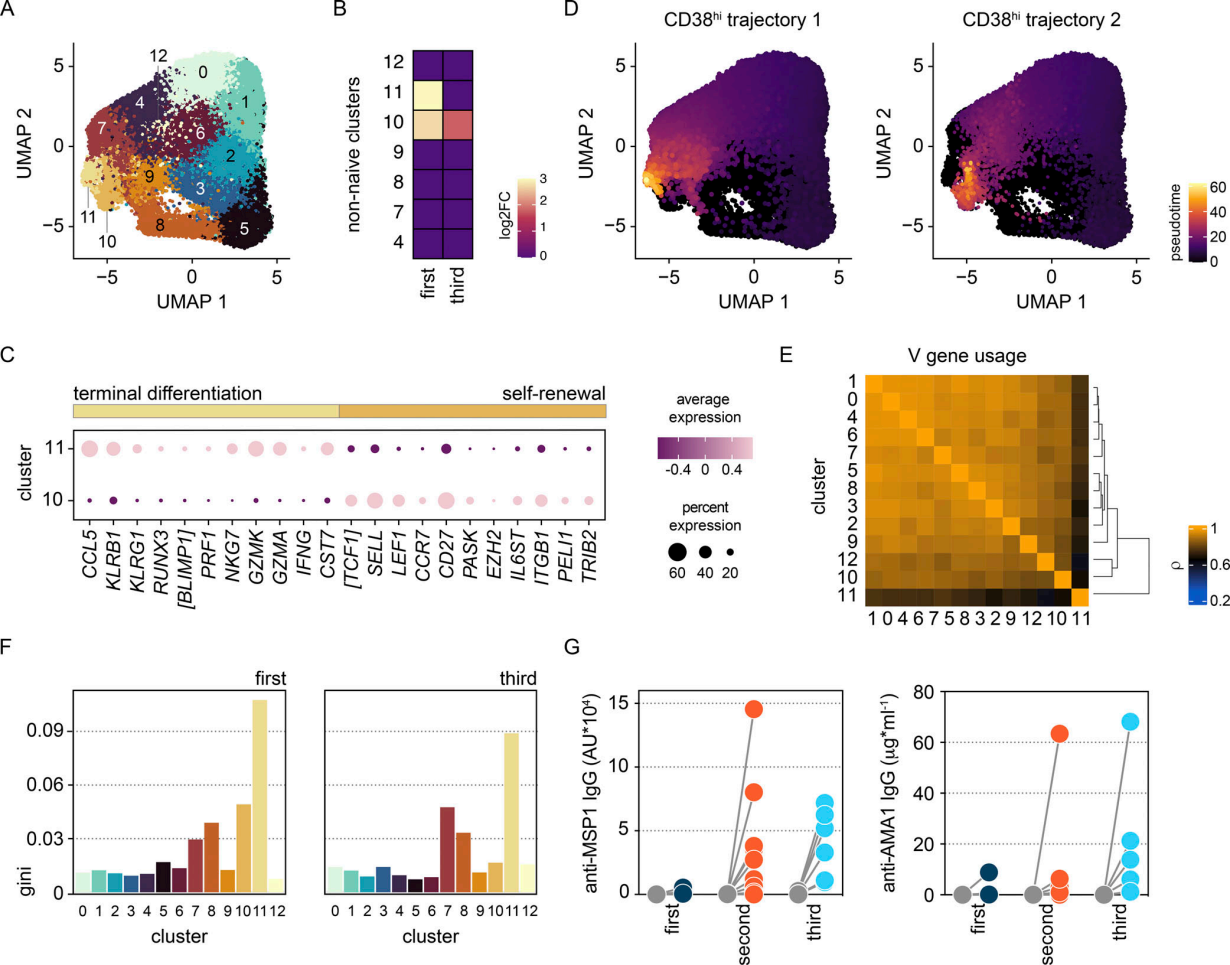
**Stem-like memory CD4** ^**+**^ **T cells respond to rechallenge.** Droplet-based single-cell RNAseq was carried out during VAC063C on flow-sorted CD4^+^ T cells obtained at baseline and T6 from volunteers undergoing their first or third infection of life. **(A)** Data from all volunteers and time points was concatenated for UMAP analysis and FlowSOM identified 13 discrete clusters of CD4^+^ T cells across the dataset (each given a unique color). **(B)** Heatmap showing the differential abundance of non-naive CD4^+^ T cell clusters at T6 (versus baseline) in first and third infection (FDR < 0.05). Note that non-significant clusters are shown with a log_2_ fold-change (FC) of zero and the identification of non-naive clusters is shown in Fig. S3 C. **(C)** Dot plot showing differentially expressed signature genes in clusters 10 and 11 (adj P < 0.05). Square brackets indicate that common protein names have been used. **(D)** Trajectory inference with Slingshot revealed clusters 10 and 11 as discrete non-overlapping endpoints of CD4^+^ T cell activation and differentiation (analysis was performed on concatenated data and CD38^hi^ cells were set as the endpoint; Slingshot identified two possible non-overlapping routes). **(E)** Spearman correlation matrix showing shared V gene usage (TRAV and TRBV) across all CD4^+^ T cell clusters (the order of features was determined by unsupervised hierarchical clustering). **(F)** Gini plot showing the equality of V gene usage in each CD4^+^ T cell cluster in the first and third infection; zero denotes perfect equality, which indicates a diverse TCR repertoire. **(G)** Class-switched antibodies (IgG) recognizing the malaria antigens MSP1 and AMA1 were quantified in serum by ELISA at baseline (grey dots) and 1 mo after challenge (colored dots). Samples were obtained from volunteers undergoing their first, second, or third infection during VAC063A, VAC063B, and VAC063C, respectively, and these data are presented in Salkeld et al. (2022). In A–F, *n* = 3 for the first and third infections, whereas in G, *n* = 10 for first and second infections and *n* = 6 in third infection.

A major limitation of bulk RNAseq is that there is very little power to detect transcriptional changes if only a small proportion of cells are responding, as seemed to be the case in the third infection. The transcriptional landscape of reactivated cells therefore remained largely unclear. To overcome this limitation, we used single-cell RNAseq to examine individual CD4^+^ T cells, which were sorted at baseline and T6 from three volunteers undergoing first infection and three undergoing third infection; samples were barcoded with oligo-tagged antibodies and superloaded onto the 10X Chromium platform (Stoeckius et al., 2018). Three libraries were then prepared and sequenced (cell surface, 5′ gene expression, and V(D)J), and after quality control (including doublet exclusion), our dataset contained ∼25,000 cells per infection with a median of 75,000 reads and 1,000 genes per cell (Fig. S3, A and B). Initially, we concatenated all data to identify the heterogeneity of CD4^+^ T cells across the dataset and uncovered 13 unique clusters (7 with a non-naive phenotype) (Fig. 5 A and Fig. S3 C). We then split the data by volunteer and time point to examine cluster abundance by linear regression and found that in third infection, a single cluster of CD4^+^ T cells (cluster 10) had expanded at T6 (Fig. 5 B). Importantly, this cluster was transcriptionally high for the canonical activation marker CD38 (Fig. S3 D).

We, therefore, examined the signature genes associated with cluster 10 and found enrichment of the transcription factor TCF1 (gene name *Tcf7*, Table S2); this has been associated with circulating T_FH_ cells in a mouse model of malaria (Lonnberg et al., 2017), but we found no significant enrichment for PD1, CXCR5, or BCL6 (Fig. S3 E). Instead, we found that cluster 10 was enriched for LEF1, SELL, TRIB2, and PELI1 (Fig. 5 C), which have all been linked to the maintenance of stem-like properties in T cells (Cao et al., 2023; Galletti et al., 2020; Ko et al., 2021). Indeed, we found that BLIMP1, whose induction is required for terminal differentiation, was repressed in cluster 10 (Fig. S3 F) and BLIMP1 is a known target of TCF1 (Choi et al., 2015; Zhao et al., 2022). Cluster 10 was also found to expand in the first infection but was exceeded by cluster 11, which was similarly high for CD38 but otherwise transcriptionally distinct (Fig. 5, B and C and Fig. S3 D). Cells in cluster 11 were enriched for signature genes associated with T_H_1 polarization, terminal differentiation, and cytotoxicity, including NKG7 and KLRB1 (CD161), which are hallmarks of rapidly responding short-lived effector cells (Mathewson et al., 2021; Ng et al., 2020). Notably, cluster 11 downregulated TCF1 and this could also be seen in our bulk RNAseq data (Fig. 4 C and Fig. S3 F).

There was no evidence, however, that cells in cluster 11 were descendants of cluster 10, and instead, trajectory analysis indicated bifurcation along the TCF1/BLIMP1 axis (Fig. 5 D and Fig. S3 G). In agreement, TCR V gene usage in cluster 11 diverged from all other T cell clusters (including cluster 10), leading to an overall reduction in repertoire diversity (Fig. 5, E and F). These data are consistent with the rapid clonal expansion of short-lived effectors in first infection, which are silenced upon rechallenge. In contrast, stem-like memory T cells increased their repertoire diversity to resemble naive T cells during the third infection (Fig. 5 F), which suggests that cluster 10 is selected for a polyclonal repertoire across multiple malaria episodes. Importantly, the maintenance of stem-like memory appears to support stepwise boosting and diversification of class-switched antibodies against parasite antigens (Fig. 5 G).

### Cytotoxic T cells are silenced for a minimum of 8 mo

The terminal differentiation of cytotoxic CD4^+^ T cells has been described in autoinflammatory disease, the tumor microenvironment, and chronic (or latent) viral infection (Appay et al., 2002; Mattoo et al., 2016; Oh et al., 2020). We were nevertheless surprised that our bulk and single-cell RNAseq data both suggested that T_H_1-polarized CD4^+^ T cells acquired transcriptional features of cytotoxicity during a first-in-life malaria episode (Fig. 4 D and Fig. 5 C). Furthermore, the scale of activation (up to 80% of all circulating cells with an effector or effector memory phenotype [Fig. 4, A and B]) was staggering and far exceeded observations made in other human challenge models of acute infection, such as influenza (Rahil et al., 2020) or typhoidal salmonella (Napolitani et al., 2018). Our data therefore suggested that rather than the response to rechallenge being unusual, the host response to a first infection was excessive and disproportionate. To explore this idea, we used mass cytometry (CyTOF) so that we could examine more cells, extend our analysis to all major T cell subsets, and quantify the heterogeneity of activated cells at the protein level. To this end, we designed an antibody panel that prioritized key markers of T cell function and fate (Table S3).

**Figure S4.**
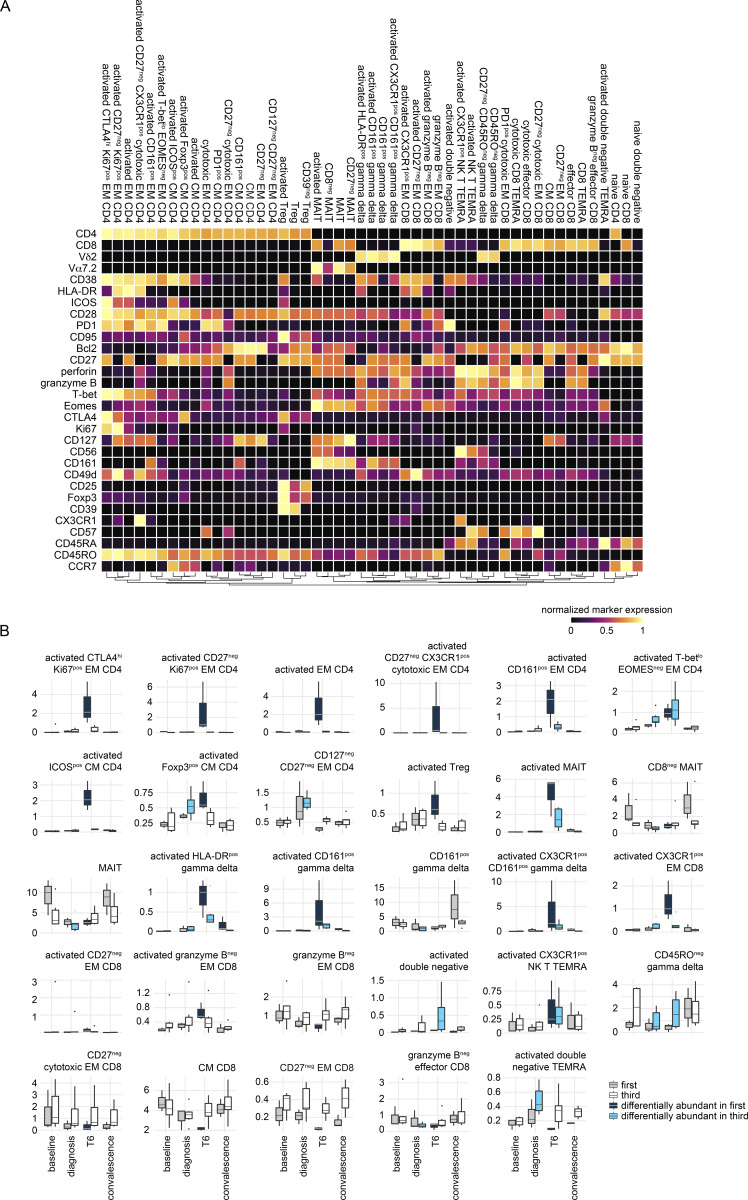
**CyTOF dynamics of T cell cluster frequencies. (A)** Heatmap showing the normalized median expression values of all markers used for clustering in each of the 49 T cell clusters identified in VAC063C. Names were assigned manually using activation, lineage, and memory markers to broadly categorize each T cell cluster; when more than one cluster was placed into the same category (e.g., activated effector memory [EM] CD4) clusters were given an accessory label to highlight their unique phenotype or property (e.g., T-bet^lo^ Eomes^neg^). The order of features was determined by unsupervised hierarchical clustering. **(B)** All T cell clusters that were differentially abundant at any time point (relative to baseline) during VAC063C. Each cluster is shown as a proportion of total CD45^pos^ CD3^pos^ T cells and clusters are shown in the same order as the heatmap in part (A) (left to right and top to bottom). Box (median and IQR) and whisker (1.5× upper or lower IQR) plots are shown (with outliers as dots) and significance (FDR < 0.05 and >2 fold-change) is indicated by color (dark blue for first infection and bright blue for third). In all plots, *n* = 3 for the first infection and *n* = 6 for the third infection. CM, central memory; MAIT, mucosal-associated invariant T cell; NK, natural killer; TEMRA, terminally differentiated effector memory cell re-expressing CD45RA; Treg, regulatory T cell.

**Figure 6.**
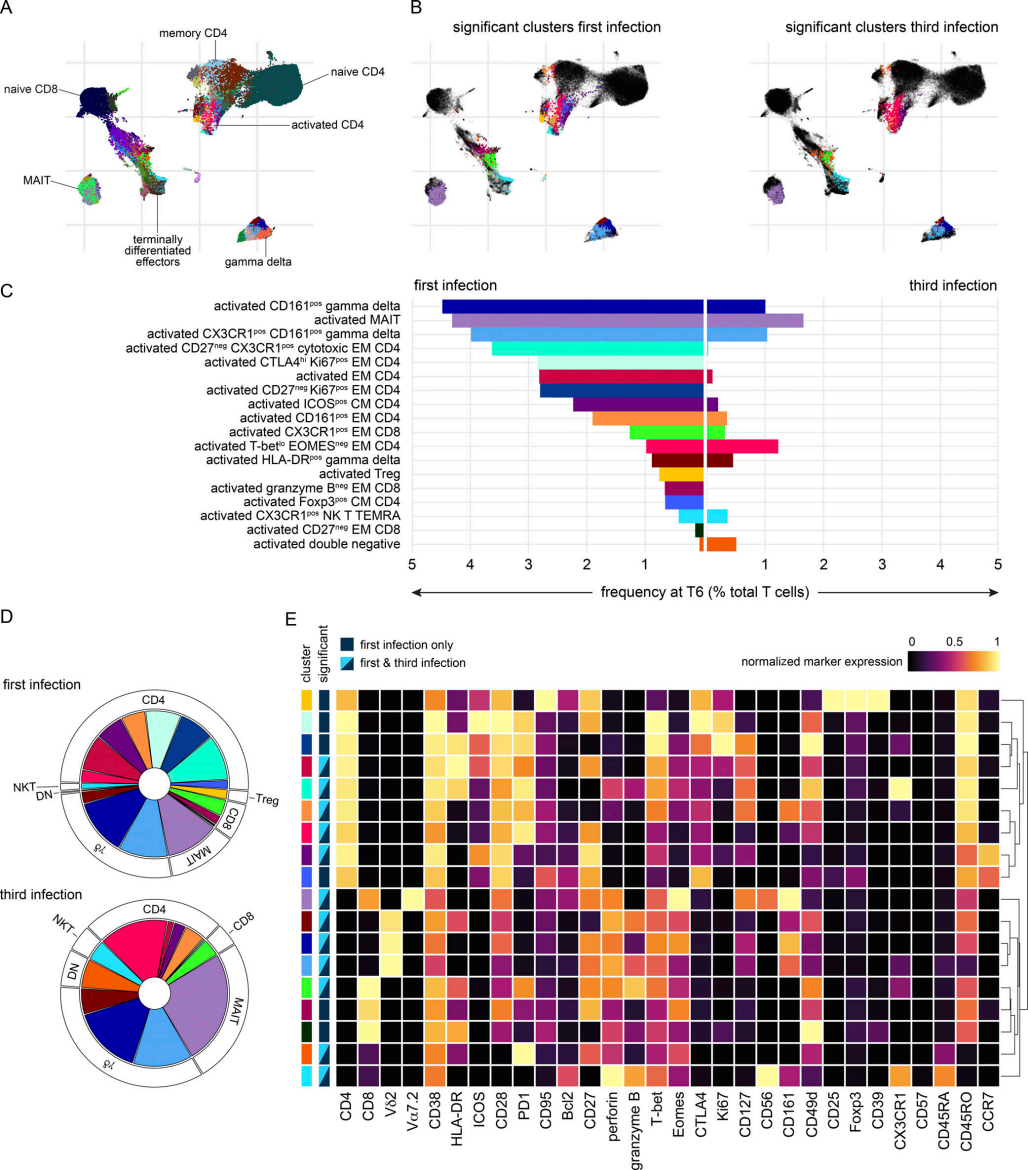
**Cytotoxic T cells are silenced for a minimum of 8 mo.** Whole blood was preserved within 30 min of blood draw at baseline, diagnosis, and T6 during VAC063C as well as 45 days after challenge (convalescence). Samples were stained with a T cell–focused antibody panel (see Table S3) and acquired on a Helios mass cytometer. After the exclusion of normalization beads and doublets, we gated on CD45^pos^ CD3^pos^ T cells for downstream steps. **(A)** Data from all volunteers and time points was concatenated for UMAP analysis and FlowSOM identified 49 discrete clusters of T cells across the dataset (each given a unique color). The major T cell subsets are labeled according to the expression of lineage, memory, and activation markers. **(B)** UMAP showing the T cell clusters that are differentially abundant at T6 (versus baseline) in the first and third infection (FDR < 0.05 and >2 fold-change). Clusters that are not significant are shown in black. **(C)** The mean frequency of each T cell cluster that is differentially abundant in first and/or third infection is shown as a proportion of all CD45^pos^ CD3^pos^ T cells at T6. CM, central memory; DN, double negative; EM, effector memory; MAIT, mucosal-associated invariant T cell; NK, natural killer; TEMRA, terminally differentiated effector memory cell re-expressing CD45RA; Treg, regulatory T cell. **(D)** Pies show the relative size of each differentially abundant cluster. **(E)** Heatmap showing the normalized median expression values of all markers used for clustering in each of the differentially abundant T cell clusters. The order of features was determined by unsupervised hierarchical clustering. Color codes to the left of the heatmap indicate cluster identity and show whether clusters were significant in the first infection or the first and third infections. Note that no cluster was unique to third infection. In A–E, *n* = 3 for the first infection and *n* = 6 for the third infection. The gap between VAC063B and C was 8 mo.

Whole blood samples were preserved in Cytodelics stabilization buffer within 30 min of blood draw at baseline, diagnosis, and T6 (as well as during convalescence) during VAC063C. As before, we concatenated all data to identify the heterogeneity of T cells across the entire dataset and uncovered 49 unique clusters (Fig. S4 A). As expected, most of the diversity was observed within the non-naive CD4^+^ and CD8^+^ T cell subsets (Fig. 6 A). Tracking the frequency of each cluster through time then resolved dynamic changes in the T cell compartment, and we performed linear regression on cell count data using edgeR (Robinson et al., 2010) to identify the differentially abundant clusters at each time point (false discovery rate [FDR] < 0.05 and absolute fold-change >2) (Fig. S4 B).

**Figure S5.**
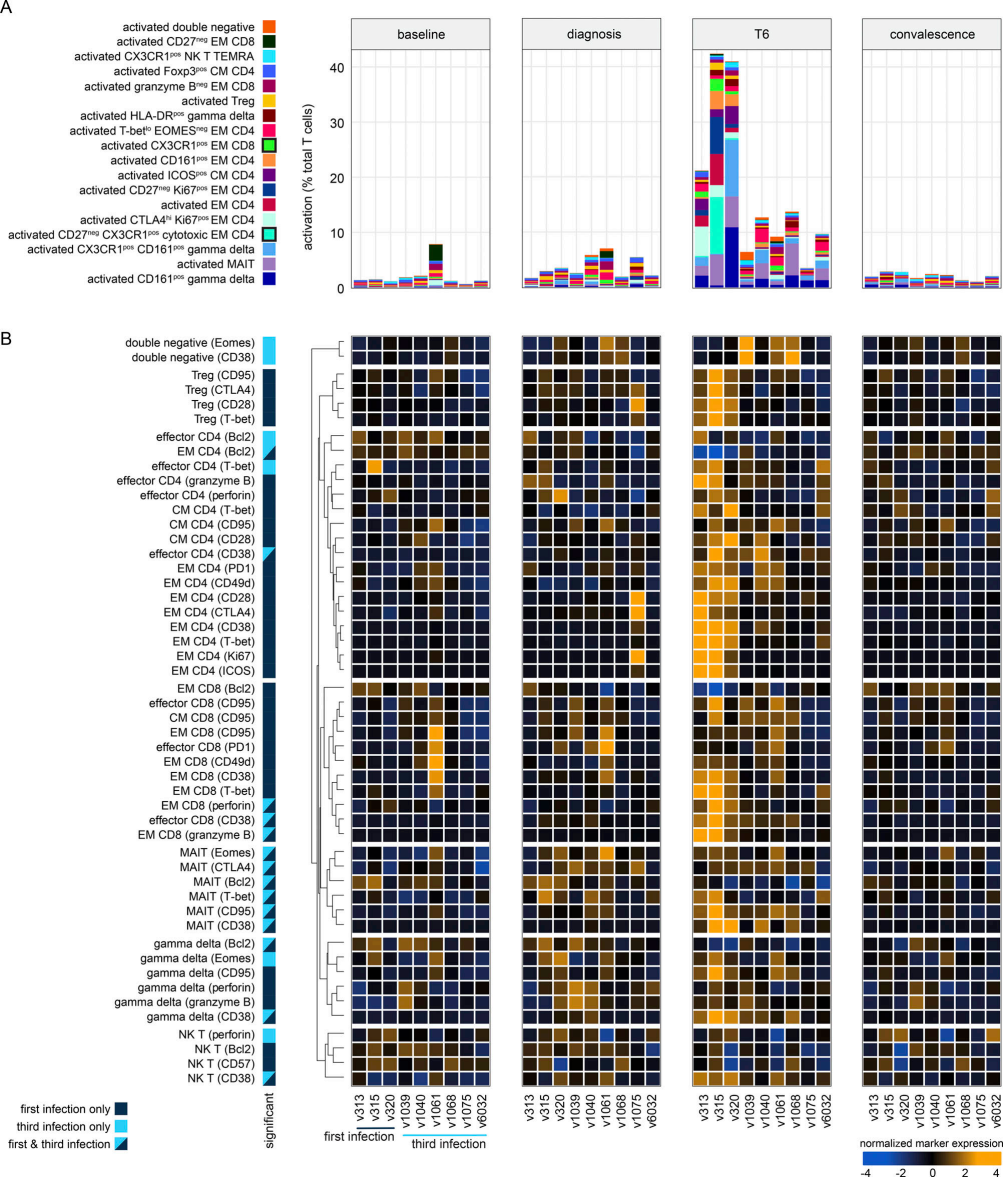
**Cytotoxic T cells are silenced after a single infection. (A)** Stacked bar chart showing the frequency of activated (CD38^hi^ Bcl2^lo^) T cells at baseline, diagnosis, and T6 as well as 45 days after challenge (convalescence) during VAC063C. Each bar represents one volunteer, and individual T cell clusters are color-coded to match Fig. 6 (note that only differentially abundant clusters are included). The major CD4^+^ and CD8^+^ T cell clusters with cytotoxic features are highlighted with a black border in the key to the left of the plot. **(B)** Differential marker expression through time in each major T cell subset. First, T cell clusters belonging to the same lineage were merged and then CD4^+^ and CD8^+^ T cells were split into naive, effector, effector memory (EM), and central memory (CM) subsets. Next, linear models were used to independently assess differential marker expression in each subset at each time point (relative to baseline); a shift in median expression of at least 10% and an FDR < 0.05 were required for significance. Shown are all subset/marker pairs that were called as significant at T6 and data are presented as row-wise z-score marker intensities. Color codes to the left of the heatmap indicate whether markers were differentially expressed during first infection, third infection or both infections. MAIT, mucosal-associated invariant T cell; NK, natural killer; TEMRA, terminally differentiated effector memory cell re-expressing CD45RA; Treg, regulatory T cell.

In the first infection, 18 T cell clusters increased in abundance at T6 and all had an activated (CD38^hi^ Bcl2^lo^) phenotype; these comprised 12 adaptive and 6 innate-like clusters that spanned every T cell lineage (Fig. 6, B–D). In sum, these clusters accounted for ∼40% of the T cell compartment (Fig. S5 A) and included cytotoxic effectors belonging to the CD8^+^, γδ, and natural killer T cell subsets—all were characterized by upregulation of the chemokine receptor CX3CR1 (Fig. 6 E). CD4^+^ T cells nevertheless dominated this response and we observed enormous heterogeneity with the expansion of nine distinct clusters. There were common traits (such as expression of the memory marker CD45RO) and most had an effector (CCR7^neg^) phenotype, but the expression of other activation and differentiation markers (inc. T-bet) was highly variable (Fig. 6 E). The largest cluster of activated CD4^+^ T cells had an unusual CD27^neg^ CX3CR1^pos^ cytotoxic phenotype and there were other unexpected features of terminal differentiation such as the appearance of perforin-expressing CD161^pos^ effectors. We, therefore, used limma to complement our analysis of cluster abundance and modeled changes in marker expression through time; this confirmed significant upregulation of both granzyme B and perforin in CD4^+^ T cells 6 days after drug treatment (Fig. S5 B). Evidently, cytotoxicity is a cardinal feature of the entire T cell response to a first-in-life malaria episode.

In third infection, the T cell response was primarily driven by activated innate-like T cells but their abundance was substantially reduced (compared with first infection) and the induction of granzyme B and perforin was suppressed (Fig. 6, C and D; and Fig. S5 B). The number of cytotoxic CD8^+^ T cells was also reduced but the most dramatic change was observed within the CD4^+^ T cell compartment. Here all but one of the nine clusters that expanded in first infection was attenuated and the previously dominant CD27^neg^ CX3CR1^pos^ cytotoxic cluster was completely silenced. These changes were not associated with an increase in Tregs (Foxp3^hi^ CD39^hi^ cells did not expand after rechallenge) or an upregulation of suppressor molecules (Fig. S5 B). In fact, only two clusters increased in size in the third compared with first infection—a small subset of double negative (DN) T cells and a cluster of T-bet^lo^ Eomes^neg^ effector memory CD4^+^ T cells (Fig. 6, C–E). The majority of activated CD4^+^ T cells belonged to this cluster in third infection, and in contrast to the first infection, these cells had already increased in abundance at diagnosis (Fig. S4 B); these presumably represent the stem-like memory cells identified by single-cell RNAseq. In sum, ∼10% of the T cell compartment was activated after rechallenge, which more closely aligns with the scale of T cell activation observed in other febrile human infectious diseases.

### Controlling T cell activation protects host tissues

**Figure 7.**
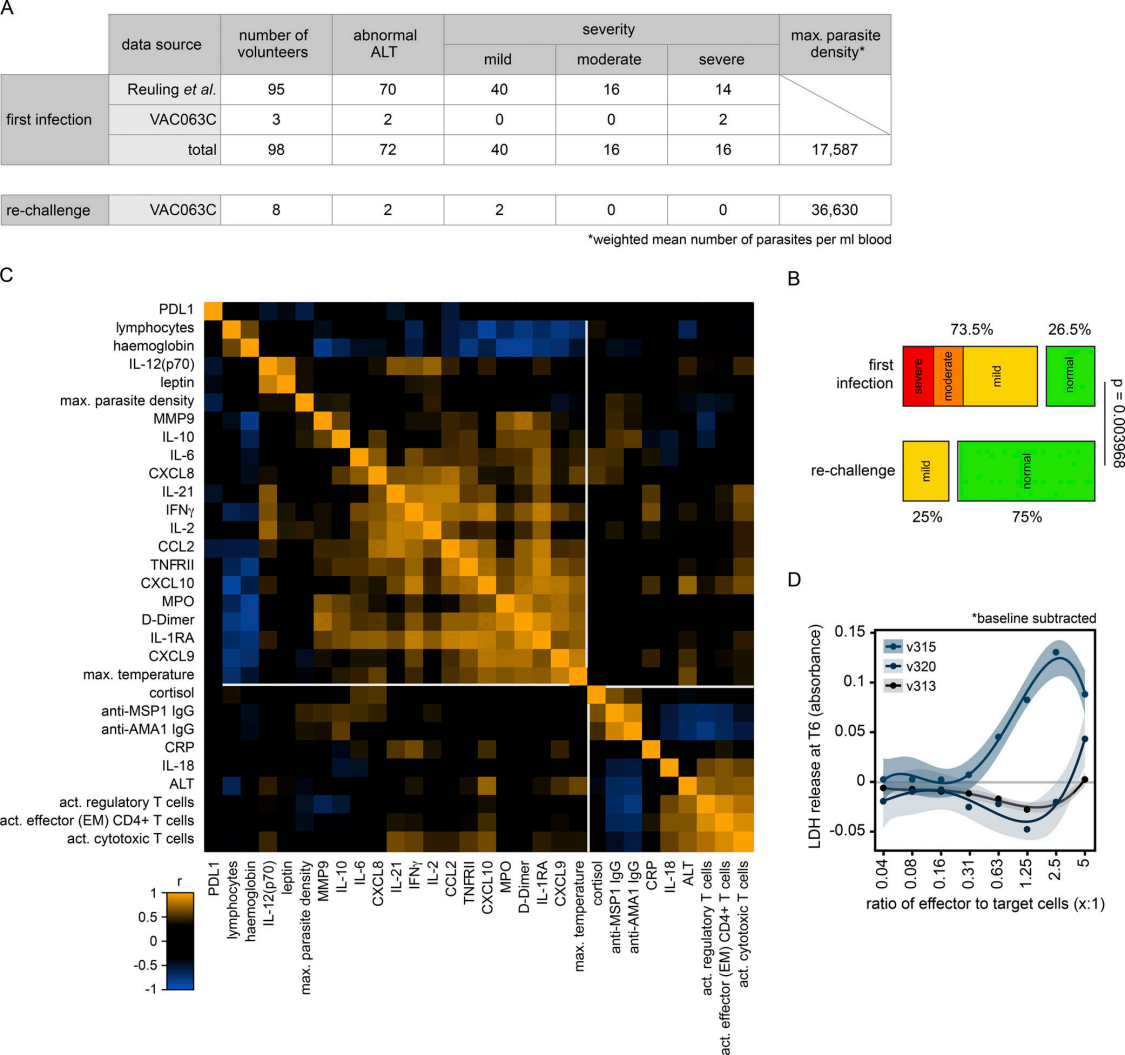
**Controlling T cell activation protects host tissues. (A)** A surrogate dataset from Reuling et al. (2018) was used to extract information on the frequency and severity of abnormal ALT during a first-in-life infection (up to 6 days after treatment). All volunteers were infected with *P. falciparum* (3D7 or NF54) as part of a CHMI trial that used equivalent end-points to our own study and in every case abnormal ALT was scored using the same adaptation of the WHO adverse event grading system (see Materials and methods). Data from 95 volunteers in Reuling et al. (those enrolled in the EHMI-3, LSA-3, EHMI-8B, EHMI-9, ZonMw2, TIP5, and CHMI-trans1 studies) and the three first infection volunteers in VAC063C were pooled for analysis. **(B)** Frequency and severity of liver injury; Barnard’s test was used to statistically determine whether an abnormal ALT reading was more prevalent during a first-in-life infection compared with the second or third infection (a P value below 0.05 was considered significant). **(C)** Pearson correlation matrix showing the fold-change of differentially abundant plasma analytes, lymphocytes, and hemoglobin during VAC063C. Fold-change was calculated either at diagnosis or T6 (relative to baseline) according to when this was largest for each feature. Also included are maximum parasite density, maximum core temperature (up to 48 h after treatment), and class-switched antibody titer (28 days after challenge). Finally, circulating ALT is shown at T6 together with the frequency of activated (CD38^hi^) effector (effector memory) CD4^+^ T cells, regulatory T cells, and cytotoxic T cells (defined as granzyme B^pos^). All data were log_2_ transformed and the order of features was determined by unsupervised hierarchical clustering. **(D)** PBMC were isolated during VAC063C from volunteers undergoing their first infection of life, restimulated in vitro with PMA/ionomycin, and cocultured with HepG2 cells for 24 h. Cytotoxicity was measured by the release of LDH. Experiments were performed using baseline and T6 samples, and data are shown as baseline subtracted values (i.e., absorbance at T6 minus absorbance at baseline). Curves were fit using a cubic polynomial function (the shaded areas represent 95% confidence intervals). Note that LDH release was shown experimentally to be specific to HepG2 cells and all assays were run in duplicate. In A and B, *n* = 98 for the first infection and *n* = 8 for rechallenge (2 second infection and 6 third infection). In C, *n* = 10 (3 first infection, 2 second infection, and 5 third infection) and in D, *n* = 3 (first infection only).

Collectively, these data show that the T cell response to a first-in-life malaria episode is dominated by heterogeneous CD4^+^ T cells that present with unusual features of terminal differentiation and cytotoxicity. To directly test whether T cell activation could be pathogenic, we measured biomarkers of collateral tissue damage. The blood-stage of infection frequently causes liver injury in naive hosts (Chughlay et al., 2020; Reuling et al., 2018; Woodford et al., 2018), and auto-aggressive T cells can directly kill primary human hepatocytes (Dudek et al., 2021). We therefore measured alanine aminotransferase (ALT) to provide a readout of hepatocellular death in vivo. In the first infection, two out of three volunteers had abnormal ALT (more than the upper limit of the reference range), which was accompanied by increased gamma-glutamyl transferase and aspartate aminotransferase, leading to moderate or severe adverse events at T6 (Salkeld et al., 2022). In contrast, there was little evidence of any deviation from baseline in liver function tests after rechallenge. To expand our sample size, we performed a meta-analysis using a previously published surrogate dataset; specifically, we examined posttreatment ALT measurements in almost 100 volunteers experiencing a first-in-life infection as part of a human challenge study (Reuling et al., 2018). Importantly, we only included CHMI trials that were directly comparable to our own rechallenge study, that is they used the same clonal parasite genotype (3D7 or the parental NF54 line), parasites had recently been mosquito-transmitted, and similar end-points were applied (treatment at around 10,000 parasites ml^−1^) (Fig. 7 A). We found that the prevalence of abnormal ALT was reduced from 75% during first infection to 25% upon rechallenge (Fig. 7 B), and in those rare cases where ALT was increased in the second or third infection, adverse events were mild (not moderate or severe) even though pathogen load was increased.

We then looked more closely at the relationship between T cell activation and liver injury in our cohort. We found a strong positive correlation between ALT and the frequency of activated effector or effector memory CD4^+^ T cells, regulatory T cells, and cytotoxic T cells (Fig. 7 C and Table S4). Of note, these all had a strong negative correlation with antibody titers. What’s more, they had no discernible relationship with markers of systemic inflammation or clinical symptoms of malaria, which were instead closely associated with each other but located in a separate clade. Finally, we cocultured peripheral blood mononuclear cells (PBMC) from the first infection with HepG2 cells (an immortalized hepatocyte cell line) and found enhanced killing at T6 (compared to baseline) in vitro (Fig. 7 D). This was particularly prominent for v315, who had the largest population of activated CD27^neg^ CX3CR1^pos^ cytotoxic CD4^+^ T cells (Fig. S5 A). Taken together, these data show that the risk of tissue damage and injury can be significantly reduced in the absence of parasite control, which provides the first in vivo evidence that long-lived mechanisms of disease tolerance operate in human malaria and can be acquired after a single infection. Moreover, protection does not require the attenuation of systemic inflammation but instead coincides with host control of T cell activation and cytotoxicity.

## Discussion

It has long been recognized that immunity to severe malaria is acquired early in life, offers protection against all manifestations of severe disease, and usually precedes clinical immunity (the transition to asymptomatic infection) by more than a decade (Doolan et al., 2009; Moxon et al., 2020; Snow and Marsh, 1998; Snow et al., 1998). We also know that immunity to severe malaria does not require improved parasite control and is thus underpinned by acquired mechanisms of disease tolerance (Gonçalves et al., 2014). The host adaptations that promote tolerance have remained unclear, in part, because of the difficulty of monitoring individuals through multiple independent infections in an endemic setting. It is here that CHMI offers a unique opportunity to track the development of disease tolerance. Furthermore, the frequency of life-threatening complications is higher in naïve adults than in children, which means we can study those with the highest risk of severe disease (Baird et al., 1998, 2003; Broderick et al., 2015; Checkley et al., 2012; Greenberg and Lobel, 1990).

Host control of inflammation could provide a rapid route to disease tolerance, but our data show that malaria parasites trigger a hardwired innate immune response across the first three infections of life. This correlates closely with symptomatology in our cohort and is consistent with repeated episodes of fever in children living in endemic areas. In contrast, T cell activation is quickly modified to limit the number and diversity of effector cells and to avoid cell fates associated with autoinflammatory disease. In turn, this limits liver damage and injury. We therefore find that T cells and tissue damage are attenuated as the risk of severe disease begins to exponentially decrease. This adaptation is likely just the tip of the iceberg, and we propose that multiple complementary strategies of disease tolerance will need to cooperate to generate immunity against severe malaria. After all, the risk of severe malaria does not decrease to zero after a single infection. Nevertheless, our data may begin to explain why large multicenter genome-wide association studies have repeatedly failed to identify immune loci that are associated with severe disease (Malaria Genomic Epidemiology Network, 2019)—variation in human T cells is almost exclusively driven by non-heritable factors (Patin et al., 2018).

As such, if we want to understand how the immune response may exacerbate disease, we should start by asking how malaria triggers such widespread and indiscriminate activation of cytotoxic T cells in a naive host. These are enriched for markers of terminal differentiation and likely represent short-lived effectors (as evidenced by the downregulation of TCF1 and de-repression of BLIMP1 in CD4^+^ T cells). What remains unclear, however, is whether these are malaria-specific cells or activated via bystander (TCR-independent) mechanisms. If the latter, these could be derived from pre-existing memory cells, which have a far lower threshold for activation than naive cells (Raué et al., 2013; Weng et al., 2002). Alternatively, malaria may activate autoreactive T cells in the same way that it can activate B cells that produce autoantibodies (Greenwood, 1974; Mourão et al., 2020). Central tolerance (which deletes self-reactive clones in the thymus) is only partially effective and there is enormous degeneracy in TCR reactivity (Welsh and Selin, 2002). Systemic infection with a pathogen carrying >5,000 protein-coding genes may therefore lead to considerable cross-reactivity between the parasite and host. In either case, the activation of bystander or cross-reactive T cells would explain our observation that the TCRβ repertoire is essentially unchanged after a first malaria episode.

And how might activation of a large heterogeneous pool of cytotoxic T cells cause harm? One possibility is that T cell activation causes extensive activation of the endothelium, leading to enhanced parasite cytoadherence and increased pathogen load (an important contributing factor for severe malaria). This has yet to be formally tested but could be assessed ex vivo using 3D models of the human microvasculature (Bernabeu et al., 2019). This is an important next step because EPCR-binding variants are known to associate with severe disease but do not have an intrinsic growth advantage in vivo (Milne et al., 2021); the host factor that promotes their selection remains to be determined. An alternative explanation is that cytotoxic T cells disrupt the endothelial barrier in critical organs and there is emerging evidence for this pathological process in children and adults with cerebral malaria (Riggle et al., 2020; Wassmer et al., 2024). Furthermore, cytotoxicity may damage parenchymal tissue, and our data support a direct role for activated T cells in hepatocyte death. In every case, the recruitment of T cells and their indiscriminate activation would be expected to drive tissue-specific inflammation, which in turn could amplify syndrome-specific disease phenotypes.

We therefore need to start asking how malaria modifies the T cell response so quickly if we want to obtain a mechanistic understanding of disease tolerance. One possibility is that infection initiates heritable epigenetic programs that reduce T cell responsiveness. Our data do not provide transcriptional evidence of anergy or exhaustion, but this hypothesis needs to be directly tested. An alternative explanation could be suppression by conventional (thymus-derived) regulatory T cells, but we found no evidence for their activation in the second or third infection. It seems that regulatory T cells are redundant at this point because there is no explosive effector response to keep in check. In much the same way, we found no evidence for the development of IL-10–producing T_R_1 cells, which have been extensively described in endemic settings (Edwards et al., 2023; Jagannathan et al., 2014; Walther et al., 2009). At first glance, it could be assumed that this is because T_R_1 cells are generated in the context of chronic stimulation and CHMI is a model of acute infection. However, even in CHMI (where drug treatment is usually initiated within 8–12 days of infection), malaria chronically stimulates the immune system. This is because once-infected red cells carry parasite-derived surface antigens and continue to circulate for weeks after drug treatment (Newton et al., 2001), follicular dendritic cells present antigens within follicles for months (possibly years) (Heesters et al., 2021), and hemozoin (an insoluble by-product of infection) persists in lymphoid tissues indefinitely (Clark and Tomlinson, 1949). Indeed, evidence of chronic stimulation can clearly be seen in our study—two clusters of activated (CD38^hi^ Bcl2^lo^) γδ T cells are still expanded 45 days after infection (Fig. S4 B). It is this persistence of parasite-derived material that we believe is responsible for the long-term protection afforded by a single malaria episode. After all, hemozoin-loaded dendritic cells have a reduced capacity to stimulate T cell proliferation in vitro (Osii et al., 2020) and a similar mechanism operating within the spleen could provide the simplest explanation for tolerance.

In much the same way, antigen-presenting cells can be modified by chronic viral infection, and this has been shown to preferentially promote the generation of memory CD8^+^ T cells with stem-like properties (Snell et al., 2018). A similar stem-like fate has recently been described for CD4^+^ T cells (Zou et al., 2024), and these appear to share remarkable overlap with the TCF1^+^ cluster that is reactivated in our cohort upon rechallenge. This is a potentially significant finding because suppression of T cells is frequently cited as a contributing factor to the slow development of antiparasite immunity in malaria. And it may appear logical that disease tolerance would come at the cost of pathogen control. Yet the activation of stem-like memory CD4^+^ T cells in third infection coincides with the boosting (MSP-1) and diversification (AMA-1) of parasite-specific class-switched antibodies, and antiparasite immunity is dependent upon diversification (not affinity maturation) (Kimingi et al., 2022; Osier et al., 2008; Rono et al., 2013). In this context, TCF1 is known to endow memory CD4^+^ T cells with the capacity for asymmetric division (Nish et al., 2017) to promote both self-renewal and the differentiation of T_FH_ precursors (Choi et al., 2015; Xu et al., 2015). The expansion of polyclonal stem-like memory CD4^+^ T cells in third infection could therefore support the selection of a broad repertoire of B cells; as such, we argue that the slow acquisition of antiparasite immunity is not a failure of T cell help.

So, do equivalent data from endemic settings support our findings? An important caveat here is that in almost every case, samples are collected around the time of drug treatment when activated T cells are still sequestered in inflamed tissues. Nevertheless, we can ascertain that circulating cytotoxic (granzyme B^pos^) CD4^+^ T cells do not expand in children or adults during an uncomplicated episode of febrile disease (Furtado et al., 2021, *Preprint*). In this study, all of these patients would be expected to have acquired immunity to severe malaria because they have lived for at least 2 years in a perennial high-transmission setting (183 infective bites per person per year). On the other hand, adults with effective antiparasite immunity have an expanded population of CD161^pos^ central memory CD4^+^ T cells (de Jong et al., 2021), which may share some similarities with the stem-like memory cells identified here. That’s because stem-like, T_CM_ and T_FH_ cells are all situated on the same branch of the TCF1-BLIMP1 axis (with cytotoxic, T_H_1, and T_R_1 cells on the other side) (Choi et al., 2015; Soon et al., 2020; Xu et al., 2015; Zhao et al., 2022). These data are therefore consistent with our findings that exposure to malaria quickly silences pathogenic T cells but does not prevent the maintenance of stem-like memory. That this can be achieved through CHMI (a short drug-cured infection) means that interventions that reduce pathogen load but don’t completely eliminate blood-stage parasites could reduce the incidence of clinical malaria in the short term and have the added benefit of providing long-lived immunity to severe malaria. Moreover, our data indicate that this protection would persist even if these interventions were stopped (drug cover or monoclonal antibodies) or lose efficacy (blood-stage vaccines).

### Limitations of the study

The absence of T6 samples during VAC063A and VAC063B necessitated a cross-sectional analysis of the T cell response to the first three infections of life and reduced our sample size to *n* = 3 for first infection. As things stand, we can be confident that malaria attenuates T cell activation after a single infection (this was reproduced in our *P. vivax* study), but we have limited insight into individual heterogeneity. It will therefore be important to repeat these findings in longitudinal studies with a larger group size. This will also give us more power to detect subtle changes in the parasite multiplication rate between first, second, and third infections and, importantly, to ask whether the kinetics of the T cell response shift forward after rechallenge. Furthermore, this will provide us with an opportunity to directly test our hypothesis that malaria triggers bystander activation of T cells during a first-in-life infection. As part of these studies, we can also examine *Plasmodium*-specific T cell responses in vitro (an important piece of the puzzle to assemble). Regardless of their specificity, it remains possible that CHMI attenuates T cell responses because of the low number of parasites inoculated and their early clearance with antimalarial drugs. We think the former is unlikely as we have previously shown that direct blood challenge does not trigger a detectable immune response until ∼24 h before diagnosis when parasitemia typically exceeds 1,000 parasites ml^-1^ (Milne et al., 2021). On the other hand, early drug treatment may be beneficial as chronic infection could prolong inflammation and in turn promote T cell exhaustion. In fact, we argue that a brief and controlled blood-stage infection would accelerate the induction of disease tolerance, and this could be assessed in endemic areas where mass drug administration programs are being expanded. However, it is important to point out that the pathological consequences of indiscriminate T cell activation during a first-in-life malaria episode remain to be examined with breadth and depth. Our data reveal enhanced killing of hepatocytes in vitro but are limited by small sample and effect sizes, as well as the inevitable drawbacks of working with immortalized cell lines. Future CHMI studies that take advantage of recent progress in human tissue sampling (for example, bone marrow, lymph node, and liver) can begin to directly address this shortfall in vivo.

## Materials and methods

### Modeling the risk of severe malaria

To examine the risk of severe disease as a function of prior exposure we used data from a Tanzanian birth cohort (Gonçalves et al., 2014). In brief, the authors examined 882 children for *P. falciparum* every 2–4 wk and recorded parasite density and disease severity for each independent infection. Data showing the incidence of severe or complicated malaria (the latter summing severe and moderately severe cases) were extracted from Fig. 2 C and Fig. S3, respectively, as shown in the published article. We then used these data to plot the total number of cases of malaria (including mild or uncomplicated episodes), impute missing values by least squares regression (as shown in Fig. S1 A), and model risk (as a function of exposure) using the fraction of severe or complicated cases divided by the total number of cases at each order of infection. Binomial regression models were then fit using maximum likelihood methods to test whether the empirical data was best explained by a constant risk (no change), a linear decrease in risk, or an exponential decay with increasing exposure. To compare model performance, we used the AIC to balance goodness of fit with model complexity and viewed a reduction in AIC > 2 as indicating a superior model. Maximum likelihood coefficients were estimated with stats4::mle() and stats::glm().

### Clinical trial design—*P*.* falciparum*

All volunteers were healthy malaria-naive adults aged between 18 and 50 years and were enrolled in up to three CHMI studies—VAC063A (November 2017), VAC063B (March 2018), and VAC063C (November 2018). The VAC063A and VAC063B trials evaluated the vaccine efficacy of the recombinant blood-stage malaria protein RH5.1 in AS01_B_ adjuvant (GSK). whereas the VAC063C trial investigated the durability of antiparasite immunity. We collected whole blood samples in each trial from the non-vaccinated (control) volunteers and analyzed 8 volunteers in VAC063A (all receiving their first infection), 10 volunteers in VAC063B (the same 8 volunteers receiving their second infection, and 2 new volunteers receiving their first infection), and 11 volunteers in VAC063C (6 of 8 from VAC063A now receiving their third infection, 2 of 2 from VAC063B now receiving their second infection, and 3 new volunteers receiving their first infection). Note that we analyzed all volunteers taking part in VAC063C and only excluded those in VAC063A and VAC063B who did not return for rechallenge. Our study design and laboratory analysis plan are shown in Table S1. All clinical trials received ethical approval from the UK National Health Service (NHS) Research Ethics Service—Oxfordshire Research Ethics Committee A for VAC063A and VAC063B (reference 16/SC/0345) and South Central Oxford A for VAC063C (reference 18/SC/0521)—and were registered at https://ClinicalTrials.gov (NCT02927145 and NCT03906474). VAC063A–C were sponsored by the University of Oxford, carried out in the UK at the Centre for Vaccinology and Tropical Medicine, and conducted according to the principles of the current revision of the Declaration of Helsinki 2008 (in full conformity with the ICH Guidelines for Good Clinical Practice). Volunteers signed written consent forms and consent was checked prior to each CHMI. Details of volunteer recruitment, inclusion/exclusion criteria, and group allocation can be found in Minassian et al. (for VAC063A and VAC063B) (Minassian et al., 2021a) and Salkeld et al. (for VAC063C) (Salkeld et al., 2022).

During each CHMI, all volunteers were infected with *P. falciparum* (clone 3D7) blood-stage parasites by direct intravenous infusion. The inoculum was thawed and prepared under aseptic conditions and volunteers received between 452 and 857 infected red cells in a total volume of 5 ml saline. Starting 1 day after challenge, volunteers attended the clinic every 12 h for assessment and blood sampling, and parasite density was measured in real-time by quantitative PCR (qPCR). The parasite multiplication rate was then calculated by fitting linear models to log_10_ transformed qPCR data, as previously described (Payne et al., 2016). In VAC063A, thick blood films were evaluated at each time point by experienced microscopists, and the diagnosis required volunteers to fulfill two out of three criteria: a positive thick blood film (one viable parasite in 200 fields) and/or qPCR data showing at least 500 parasites ml^−1^ blood and/or symptoms consistent with malaria. In VAC063B and C, microscopy was dropped to minimize the variation in parasitemia that was observed between volunteers at diagnosis; importantly, this protocol change did not impact volunteer safety, and the average parasitemia at diagnosis remained comparable with VAC063A. The new criteria for diagnosis were asymptomatic with any qPCR result above 10,000 parasites ml^−1^ or symptomatic with a qPCR result above 5,000 parasites ml^−1^. In all cases, volunteers were treated with artemether and lumefantrine (Riamet), except where its use was contraindicated and atovaquone and proguanil (Malarone) were given instead. In our analysis, we refer to the blood sample taken immediately before drug treatment (<30 min) as the diagnosis time point.

Clinical symptoms of malaria (malaise, fatigue, headache, arthralgia, back pain, myalgia, chills, rigor, sweats, nausea, vomiting, and diarrhea) were recorded by volunteers on diary cards or during clinic visits. All symptoms were recorded as adverse events and assigned a severity score 0 (absent), 1 (transient or mild discomfort), 2 (mild to moderate limitation in activity), or 3 (severe limitation in activity requiring assistance). Pyrexia was scored as absent (≤37.5°C), mild (37.6–38.2°C), moderate (38.3–38.9°C), or severe (≥39°C). Full blood counts and blood chemistry (including electrolytes, urea, creatinine, bilirubin, ALT, alkaline phosphatase, and albumin) were evaluated at the Churchill and John Radcliffe Hospital in Oxford.

### Clinical trial design—*P*.* vivax*

In VAC069A, six healthy malaria-naive adults were enrolled to test the infectivity of a new cryopreserved stabilate containing a clonal field isolate of *P. vivax* (PvW1), which had been carefully prepared for use in CHMI (Minassian et al., 2021b). Volunteers were infected with blood-stage parasites by direct intravenous infusion (as above), and the immune response to PvW1 was comprehensively characterized and compared to *P. falciparum* (3D7) (Bach et al., 2023). In VAC069B, three of these volunteers returned for a homologous rechallenge (8 mo after VAC069A) and two additional malaria-naive adults received their first challenge with PvW1. In both clinical trials, treatment was initiated once two diagnostic criteria were met: a positive thick blood smear, >5,000 or 10,000 parasites ml^−1^ blood, and/or symptoms consistent with malaria. Treatment consisted of artemether and lumefantrine (Riamet) or atovaquone and proguanil (Malarone) if Riamet was contraindicated. Whole blood sampling, processing, and downstream analyses were performed analogously to the VAC063 trials (see below). VAC069A and B were sponsored by the University of Oxford, received ethical approval from the UK NHS Research Ethics Service—South Central Hampshire A (reference 18/SC/0577)—and were registered at https://ClinicalTrials.gov (NCT03797989). The trials were conducted in line with the current version of the Declaration of Helsinki 2008 and conformed with the ICH Guidelines for Good Clinical Practice.

### Processing whole blood for RNA and plasma

Venous blood was drawn into K_2_EDTA-coated vacutainers (#367835; BD). To preserve RNA for transcriptional analysis, 1 ml of whole blood was mixed thoroughly with 2 ml Tempus reagent (#4342792; Thermo Fisher Scientific) and samples were stored at −80°C. No more than 2 h passed between the blood draw and RNA preservation. To obtain platelet-depleted plasma, 3 ml of whole blood was divided into two 2-ml Eppendorf tubes and centrifuged at 1,000 × *g* for 10 min (at 4°C) to pellet the cellular components. Working on ice, plasma was then carefully transferred to a new 2-ml tube and centrifuged at 2,000 × *g* for 15 min (at 4°C) to pellet platelets. Cell-free platelet-depleted plasma was aliquoted into 1.5-ml Eppendorf tubes, snap-frozen on dry ice, and stored at −80°C.

### Whole blood RNAseq

RNA was extracted from whole blood using the Tempus spin RNA isolation reagent kit (#4380204; Thermo Fisher Scientific) according to the manufacturer’s instructions. To account for the reduced starting volume and to maintain Tempus stabilizing reagent at the correct final concentration, we added just 1 ml PBS to each sample after thawing. Diluted samples were then centrifuged at 3,000 × *g* for 30 min (at 4°C) to pellet nucleic acids. Pellets were resuspended in RNA purification resuspension solution and centrifuged on a silica column to remove non-nucleic acid contaminants. After washing, the column was incubated for 2 min at 70°C before eluting nucleic acids. 40 μl eluate was then subjected to DNA digestion using the RNA clean and concentrator-5 kit (#R1013; Zymo Research). Purified RNA was eluted using 30 μl DNase/RNase-free water, quantified using a Qubit Fluorometer (HS RNA assay kit, #Q32852; Thermo Fisher Scientific), and RNA integrity was assessed using an Agilent Bioanalyzer 2100 (RNA 6000 nano kit, #5067-1511; Agilent). The average RNA integrity number (RIN) was 9 (98% of samples >8, lowest RIN 7). Libraries were prepared by Edinburgh Genomics (United Kingdom) using the TruSeq stranded mRNA library prep kit (#20020595; Illumina). Stranded library preparation allows transcript expression to be estimated more accurately; in particular, it is more effective in quantifying antisense gene expression, properly assigning transcripts to putative coding genes, and resolving ambiguity in reads from overlapping genes. Libraries were sequenced using the NovaSeq 6000 Illumina platform yielding 50 bp paired end (PE) reads. These short reads are sufficient to accurately capture gene expression thanks to the well-annotated human transcriptome. The average number of reads per sample passing quality control (QC) across all samples was 8.45 × 10^7^.

### Data analysis of whole blood RNAseq

The quality and content of FASTQ files, which contain raw PE sequencing reads, were assessed using FastQC (https://www.bioinformatics.babraham.ac.uk/projects/fastqc). A single sample (volunteer 1040 baseline second infection) failed quality control and we therefore excluded all RNAseq data from this volunteer’s second infection during analysis. TruSeq primer sequences were removed using Cutadapt v1.9, reads were aligned to the Ensembl release 96 *Homo sapiens* transcript set with Bowtie2 (Langmead and Salzberg, 2012) v2.2.7 (parameters —very-sensitive -p 30 —no-mixed —no-discordant —no-unal) and reads that mapped to globin transcripts were discarded (on average 11.7% of mapped reads, range 1.6–38.1%). We opted for bioinformatic globin depletion as it is highly sensitive and reproducible whereas depleting globin during RNA preparation can compromise the amount and quality of RNA recovered (Harrington et al., 2020; Shin et al., 2014). Following these steps, the average alignment rate to the human transcriptome across all samples was 88%. A matrix of normalized counts for each transcript was obtained from sorted and indexed BAM files using Samtools idxstats (https://www.htslib.org/doc/samtools.html). Transcript counts were imported into the R/Bioconductor environment (v3.6), and differential gene expression analyses (pairwise group comparisons) were performed using functions within the DESeq2 (Love et al., 2014) package. lfcShrink was applied to the output of each pairwise comparison (type normal) and lists of differentially expressed transcripts were subsequently analyzed in R. Non-coding transcripts were removed and multiple transcripts annotated to the same gene were consolidated by keeping the transcript with the highest absolute fold-change. Only protein-coding transcripts with an adjusted P value (adj P) <0.05 and a fold-change >1.5 were considered differentially expressed. Volcano/radar/dot plots and heatmaps were generated using the ggplot2 (Wickam, 2016) package. The gene lists used for cell cycle analysis were manually compiled from published datasets (Mizuno et al., 2009; Peña-Diaz et al., 2013), and we allocated genes to a single phase based on cell cycle transcriptional network analysis (Dominguez et al., 2016; Giotti et al., 2017).

### Functional gene enrichment analysis using ClueGO

Lists of differentially expressed genes were imported into ClueGO (Bindea et al., 2009; Mlecnik et al., 2014) v2.5.7. ClueGO identified significantly enriched GO terms (Biological Process and Molecular Function) associated with these genes and placed them into a functionally organized non-redundant gene ontology network based on the following parameters: adj P cutoff = 0.01; correction method used = Bonferroni step down; min. GO level = 5; max. GO level = 11; number of genes = 3; min. percentage = 5; GO fusion = true; sharing group percentage = 40; merge redundant groups with >40% overlap; kappa score threshold = 0.4; and evidence codes used [All]. Each of the functional groups was assigned a unique color, and a network was then generated using an edge-weighted spring-embedded layout based on kappa score—groups were named by the leading GO term (lowest adj P with min. GO level 5 or 6). Merged networks were constructed by inputting two lists of differentially expressed genes; for each GO term, information on what fraction of associated genes were derived from each list was retained. Any GO term containing >60% associated genes from a single list was considered to be enriched in that group, otherwise, GO terms were considered to be shared.

### Multiplexed plasma protein analysis

The concentration of 39 analytes was measured in plasma samples collected at baseline, during infection, diagnosis, 6 days after drug treatment (T6), and 28 or 45 days after the challenge (convalescence). Plasma was thawed on ice and centrifuged at 1,000 × *g* for 1 min (at 4°C) to remove potential protein aggregates. We customized four LEGENDplex panels from BioLegend and performed each assay on filter plates according to the manufacturer’s instructions. Samples and standards were acquired on an LSRFortessa flow cytometer (BD) and FCS files were processed using LEGENDplex software (version 7.1), which automatically interpolates a standard curve using the plate-specific standards and calculates analyte concentrations for each sample. All samples from v1040 were excluded after failing QC, and downstream data analysis was performed in R (v3.6). To determine which plasma proteins varied significantly through time, we used the lme4 package to fit a separate linear mixed-effects model for each analyte. All available time points were included and models were fit to log_2_ transformed data with time point as a categorical fixed effect and volunteer as a random effect. A Kenward–Roger approximation was used to calculate P values (using the pbkrtest package), which were adjusted for multiple testing using the Benjamini–Hochberg method. Results were visualized with ggplot2, and analytes with at least a 1.5 fold-change from baseline to diagnosis or T6 are shown. To determine which plasma proteins varied between the first infection and rechallenge, we used lme4 to fit mixed-effects models that included time point and infection number as categorical fixed effects (with volunteer as a random effect). In this case, linear hypothesis testing was performed using multcomp’s glht function (with Benjamini–Hochberg correction for multiple testing). Only analytes that were significant in both the second and third infection (versus first infection) are shown (adj P < 0.05 and fold-change >1.5).

### Flow-sorting CD4^+^ T cell subsets

We flow-sorted CD4^+^ T cell subsets for bulk RNAseq from 3 ml whole blood collected in K_2_EDTA vacutainers. After red cell lysis (erythrocyte lysis buffer, #00-4300-54; eBioscience), leukocytes were washed in PBS supplemented with 2% fetal bovine serum (heat inactivated and 0.22 μm filtered FBS Premium Plus, #16000044; Gibco) containing 5 mM EDTA (#AM9260G; Life Technologies). We then blocked Fc receptors (human TruStain FcX, #422302; BioLegend) and incubated leukocytes for 20 min (at 4°C) with the following fluorophore-conjugated antibodies: CD3 (clone OKT3), CD4 (clone OKT4), CD127 (clone A019D5), CD25 (clone M-A251), CCR7 (clone G043H7), CD45RA (clone HI100), CD38 (clone HIT2), and HLA-DR (clone L243) (all from BioLegend). Cells were washed with cold PBS (containing 2% FBS and 5 mM EDTA) and filtered through a 40-μm cell strainer (#352340; BD) immediately before sorting. We used a FACSAria III or Fusion cell sorter with a 70-μm nozzle running FACS Diva v8 software (sort setting = purity) to simultaneously sort 10,000 naive (CD127^pos^ CCR7^pos^ CD45RA^pos^), regulatory (CD25^hi^ CD127^neg^), and effector or effector memory (CD127^pos^ CCR7^neg^ CD45RA^neg^) CD4^+^ T cell subsets. Cells were sorted directly into RNase-free sterile 1.5 ml screw-cap tubes (#11529924; Thermo Fisher Scientific) containing 1 ml TRIzol Reagent (#15596026; Thermo Fisher Scientific) and incubated for 5 min at room temperature. Samples were then stored at −80°C. Note that sort purity was >95% for every sample, and this was assessed in real-time by simultaneously sorting naive CD4^+^ T cells into cold PBS (containing 2% FBS and 5 mM EDTA) and reacquiring these on the cell sorter.

### Bulk RNAseq of CD4^+^ T cell subsets

RNA was extracted using a modified phenol–chloroform protocol (Chomczynski and Sacchi, 2006) with 1-bromo-3-chloropropane (#B62404; Sigma-Aldrich) and isopropanol (#423835000; Acros Organics). Total RNA was quantified and integrity assessed using an Agilent Bioanalyzer 2100 (RNA 6000 pico chip, #5067-1513; Agilent); all sequenced samples had a RIN value above 8. cDNA was generated from 1 ng total RNA using the SMART-Seq v4 ultralow input RNA kit (#634894; Takara Bio) and amplified using 12 cycles of PCR. Amplified cDNA was purified using AMPure XP beads (#A63880; Beckman Coulter) and quantified on a Qubit Fluorometer (dsDNA high sensitivity kit, #Q32851; Thermo Fisher Scientific). The quality of the amplified cDNA was then assessed by a Bioanalyzer (DNA high sensitivity kit, #5067-4626; Agilent). Libraries were constructed from 150 pg cDNA using the Nextera XT DNA library preparation kit (#FC-131-1024; Illumina) according to the manufacturer’s instructions. As above, libraries were quantified by Qubit, and the quality was assessed by a bioanalyzer to measure the fragment size distribution. Using this information, samples were combined to create equimolar library pools that were sequenced on a NextSeq 550 Illumina platform to yield 75 bp PE reads; the average number of PE reads per sample passing QC across all samples was 2.74 × 10^7^.

### RNAseq analysis of CD4^+^ T cell subsets

Quality and content of FASTQ files were assessed using FastQC and reads were aligned to the Ensembl release 96 *Homo sapiens* transcript set with Bowtie2 v2.2.7 (parameters —very-sensitive —p 30 —no-mixed —no-discordant —no-unal). Our median alignment rate was 66.4% after removing primer and adapter traces (SMART-Seq v4 primers, polyG, polyT, and polyN) with Cutadapt v1.9. The sorted, indexed BAM files were then used to obtain a matrix of normalized counts for each transcript using Samtools idxstats. Transcript counts were imported into the R/Bioconductor environment (v3.6), and differential gene expression analyses (pairwise group comparisons) were performed using functions within the DESeq2 package. lfcShrink was applied to the output of each pairwise comparison (type normal), and lists of differentially expressed transcripts were filtered by removing all non-coding transcripts and retaining only those with an adj P < 0.05 and fold-change >1.5. Multiple transcripts annotated to the same gene were consolidated by keeping the transcript with the highest absolute fold-change. Heatmaps and circular stacked bar plots were generated using the ggplot2 package.

### Cryopreservation of PBMC

PBMC were isolated from 5 ml whole blood, which was diluted with an equal volume of Dulbecco’s phosphate buffered saline (D-PBS) containing 2% fetal bovine serum (FBS) (#07905; STEMCELL Technologies) and layered onto 3.5 ml Lymphoprep in a 15-ml SepMate tube (#07851 and #07905; STEMCELL Technologies). SepMate tubes were centrifuged at 1,200 × *g* for 10 min (at room temperature), the upper plasma-containing layer was discarded, and the remaining supernatant above the insert (which contains the PBMC) was poured into a 15-ml tube. Cells were then washed twice in D-PBS containing 2% FBS and cell concentration was determined using a CASY cell counter. Finally, PBMC were resuspended in FBS (heat inactivated and 0.22 μm filtered FBS Premium Plus, #16000044; Gibco) containing 10% dimethyl sulfoxide (DMSO, #D8418; Sigma-Aldrich) at a concentration of 5 × 10^6^ cells ml^−1^ and transferred to cryovials. These were placed in a Corning CoolCell freezing container, which was subsequently placed at −80°C. After 24 h, PBMC-containing cryovials were transferred to liquid nitrogen where they were stored long term. To prepare cryopreserved PBMC for use in experiments, cryovials were quickly thawed in a 37°C water bath and diluted by adding 10x volume thawing media (RPMI 1640 supplemented with 10% FBS and 20 U ml^−1^ DNase I [#D4513; Sigma-Aldrich]), one drop at a time with continuous agitation. Cells were centrifuged at 350 × *g* for 10 min (at room temperature) and washed twice in thawing media. After washing, viable cell counts were calculated using a hemocytometer.

### TCR repertoire sequencing

We analyzed the TCRβ repertoire of the six volunteers who underwent third infection during VAC063C, assessing their bulk repertoire prior to challenge (baseline) and 28 days after challenge (convalescence) during their first (VAC063A) and second (VAC063B) malaria episode. RNA was extracted from thawed PBMC using the Quick-RNA miniprep plus kit (Zymogen) as per the manufacturer’s instructions, including DNase treatment. A modified protocol from Mamedov et al. (2013) was then used to synthesize cDNA, which was treated with 1 μl Uracyl DNA glycosylase (5 U μl^−1^). A nested PCR then generated TCRβ V region amplicons with outer primers using indexed forward primers composed of the SMART synthesis oligo sequence fused to a P7 Illumina tag (5′-CAAGCAGAAGACGGCATACGAGATXXXXXXGGCGAAGCAGTGGTATCAACGCAGAGT-3′) and a reverse primer within the TCR C region fused to a P5 Illumina tag (5′-AATGATACGGCGACCACCGAGATCTACACACACSTTKTTCAGGTCCTC-3′). Library QC was performed by Nanodrop and Bioanalyzer, and asymmetric 400 + 100 bp sequencing was then performed on an Illumina NovaSeq using custom read primers (5′-ACT​CTG​CGT​TGA​TAC​CAC​TG-3′ index with 5′-CGAGATCTACACACACSTTKTTCAGGTCCTC-3′ for read 1 and 5′-GGC​GAA​GCA​GTG​GTA​TCA​ACG​CAG​AGT-3′ for read 2). The average number of functional  TCR β variable (TRBV) reads was 2.58 × 10^6^ per sample resulting in an average of 13,410 unique CDR3 sequences per sample. Quality control was performed on the raw FASTQ files using FastQC and sequencing data that incorporated unique molecular identifiers (UMI) was demultiplexed using MiGEC (Shugay et al., 2014). All sequences were aligned using MiXCR (Bolotin et al., 2015) software utilizing IMGT (Lefranc et al., 1999) nomenclature. MiGEC and MiXCR standard error correction thresholds were used (including a minimal nucleotide quality score of 20 within the target gene region) and only in-frame functional CDR3 sequences were included in downstream analyses. Custom pipelines of Python scripts were then used to analyze and plot the MiXCR output, and proportional TRBV gene usage was calculated for each sample. Note that for visualization, TRBV genes that had a zero count at any given time point were replaced with the minimum gene count prior to log_10_ transformation.

### Single-cell RNAseq of CD4^+^ T cells

Cryopreserved PBMC from volunteers 313, 315, and 320 (first infection), and 1,061, 1,068, and 6,032 (third infection) were prepared 2 days before infection (baseline) and 6 days after treatment (T6) during VAC063C. To undertake single-cell RNAseq, these samples were thawed, washed, counted, and resuspended at a concentration of 3 × 10^6^ cells ml^−1^ in PBS supplemented with 2% FBS (heat inactivated and 0.22 μm filtered FBS Premium Plus, #16000044; Gibco) and 5 mM EDTA (#AM9260G; Life Technologies). Cells were then stained with surface antibodies exactly as described above (see Flow-sorting CD4^+^ T cell subsets) but with the addition of TotalSeq-C oligo-tagged barcoding antibodies that recognize CD298 and β2-microglobulin (a combination of clones LNH-94 and 2M2, available from BioLegend). For each sample, we flow-sorted 300,000 CD4^+^ T cells (CD3^pos^ CD4^pos^) into 15-ml conical tubes containing 5 ml RPMI 1640 supplemented with 10% FBS Premium Plus. At the same time, we sorted an equal number of CD4^+^ T cells into 5 ml cold PBS (containing 2% FBS and 5 mM EDTA) and reacquired these on the cell sorter to check purity, which was >95% for every sample.

After sorting and purity checks, six individually barcoded samples were pooled (one pool for the first infection and a separate pool for third infection), and the concentration of each pool was carefully measured and adjusted to 1,262 cells µl^−1^. Each pool was then loaded into 2 wells of an A Chip, which itself was loaded onto a 10X Genomics Chromium Controller. Note that we intentionally superloaded the controller to capture ∼30,000 singlets per pool based on the workflow described in Stoeckius et al. (2018). Captured cells were then tagged with 10X cell barcodes in gel beads in emulsion (GEMs), cDNA was amplified, and libraries were constructed according to the manufacturer’s instructions (Chromium single-cell V(D)J reagent kit with feature barcoding technology for cell surface protein—protocol CG000186 revision C). From each GEM, three libraries were constructed and separately indexed: (1) the cell surface barcode, (2) 5′ gene expression, and (3) the TCR ⍺ and β chains (after amplification of the V(D)J regions). Following QC and quantification (using the Agilent Bioanalyzer and TapeStation as well as Qubit), the libraries were pooled at a ratio of 8% cell surface, 84% gene expression, and 8% V(D)J and then sequenced on a NovaSeq 6000 Illumina platform to yield at least 66,000 150 bp PE reads per cell. In total, we obtained 5,931 million PE reads (2,848 and 3,083 million PE reads for the first and third infection, respectively) of which 487 million PE reads (8.2%) were derived from the cell surface library, 4,858 million PE reads (81.9%) from the 5′ gene expression library and 586 million PE reads (9.9%) from the V(D)J library.

### Single-cell RNAseq analysis

Cell Ranger multi (v6.0.2) was used to align 5′ gene expression and V(D)J sequencing reads to the GRCh38 reference genome. This initial step was performed independently for first and third infection since both pools used the same set of six TotalSeq-C barcodes—C0251 (5′-GTC​AAC​TCT​TTA​GCG-3′), C0252 (5′-TGA​TGG​CCT​ATT​GGG-3′), C0253 (5′-TTC​CGC​CTC​TCT​TTG-3′), C0254 (5′-AGT​AAG​TTC​AGC​GTA-3′), C0255 (5′-AAG​TAT​CGT​TTC​GCA-3′), and C0256 (5′-GGT​TGC​CAG​ATG​TCA-3′) (all BioLegend). Feature-barcode matrices were then loaded into R (v4.2.0) using Seurat (v4.3.0), and oligo count matrices were generated from the cell surface library using CITE-seq-Count (v1.4.2) (https://doi.org/10.5281/zenodo.2590196, Roelli et al., 2019). These datasets were filtered to only include 10X cell barcodes that were detected in both the 5′ gene expression and cell surface libraries, and after normalization and scaling, principal components analysis (PCA)-initialized t-distributed stochastic neighbor embedding was used to visualize the data (first seven principal components [PC], perplexity = 100). For each pool of samples, an inspection of the oligo expression levels revealed six large clusters that corresponded to single positive cells (or singlets). As such, we could use nearest neighbor identification (PCA 1–6) and Louvain clustering to assign each singlet to a sample and exclude cells with multiple oligo tags (doublets). At this stage, the data from the first and third infections were combined, and a standard Seurat workflow was followed for QC. Briefly, we excluded cells with >2,500 feature counts (or <200) and cells with >5% mitochondrial transcripts. Data were log normalized and the top 2,000 most variable features were selected (after variance stabilization) for computing the PC. Harmony (Korsunsky et al., 2019) (v0.1.0) was used to integrate data and reduce individual variation, and Seurat’s wrapper for the Louvain algorithm (FindClusters) was used to cluster cells based on the first 12 PC (with default settings at a resolution of 0.8). An initial pass of this analysis identified a persistent and hyperexpanded cluster of TRBV11-3^pos^ CD4^+^ T cells. These cells were unique to v1068 (the only CMV seropositive volunteer) and did not respond to malaria; we therefore excluded this cluster from all downstream steps to avoid confounding signatures of malaria-induced activation.

Next, differential cluster abundance was modeled using negative binomial regression with glm.nb() from the MASS package. Time point was fit as a categorical variable, P values were adjusted (Benjamini–Hochberg method) and an FDR < 0.05 was considered significant. Slingshot (Street et al., 2018) was then used to perform pseudotime analysis on Harmony-derived PC (default settings); the leaf node was set to either cluster 10 or 11 (CD38^hi^ cells), but no trajectory passed through both clusters. Finally, to analyze TCR diversity, we used createHTOContigList() to filter and match Cell Ranger’s annotations on the assembled contigs to those extracted from the scRepertoire (Borcherding et al., 2020) (v1.8.0). V gene usage (TRAV and TRBV) was analyzed using standard immunarch (v0.9.0) (https://github.com/immunomind/immunarch) workflows (excluding ambiguous assignments), and the amino acid sequences of the TCR in each cluster were used to calculate their Gini coefficient (using convenience functions). Data were visualized using ComplexHeatmap (Gu et al., 2016) or ggplot2.

### Processing whole blood for mass cytometry

Venous blood was collected in K_2_EDTA-coated vacutainers, stabilized within 30 min of blood draw in whole blood stabilization buffer (#hWBCS002; Cytodelics), and stored at −80°C. For antibody staining, samples were quickly thawed in a 37°C water bath and then fixed and red cell lysed for 15 min using a whole blood preservation kit (#hC002; Cytodelics). Next cells were permeabilized with Maxpar barcode permeabilization buffer (#201057; Fluidigm), and each sample was barcoded using Cell-ID 20-plex palladium barcodes (#201060; Fluidigm). Samples were then pooled and stained with our T cell–focussed surface antibody mix (see Table S3) for 30 min. After washing, cells were fixed and permeabilized with the Maxpar nuclear antigen staining buffer set (#201063; Fluidigm) and incubated with the nuclear antibody mix for 45 min. Cells were then washed and fixed for 10 min in 1.6% formaldehyde diluted in PBS (#28906; Thermo Fisher Scientific). After a final round of washes, cells were resuspended at a concentration of 3 × 10^6^ cells ml^−1^ in 72.5 nM Cell-ID Intercalator Ir solution (#201192A; Fluidigm) and stored overnight at 4°C. Samples were acquired the next day on a freshly tuned Helios mass cytometer (acquisition rate 300-500 events per second) using the WB injector and 10% EQ four-element calibration beads (140Ce, 151Eu, 165Ho and 175Lu, #201078; Fluidigm).

### Mass cytometry data analysis

The Fluidigm CyTOF software (version 6.7) generated FCS files, which were normalized (Finck et al., 2013) and debarcoded (Zunder et al., 2015) using the R package CATALYST (Crowell et al., 2020). Samples were compensated using single stained beads (Chevrier et al., 2018). After the exclusion of normalization beads and doublets, we gated on CD45^pos^ CD3^pos^ T cells using the Cytobank web portal (https://www.cytobank.org). We then inspected the intensity distribution of each channel and removed those with low variance (CD16, CD69, CXCR5, GATA3, RORγt, TCRγδ, and TIM3). The remaining 30 markers were used for Uniform Manifold Approximation and Projection (UMAP) and FlowSOM clustering. UMAP (McInnes et al., 2020, *Preprint*) was used to generate a 2D projection of this high-dimensional dataset. Here, phenotypic similarity of cells within and between populations is preserved in the Euclidean distance of the projection. We used its R implementation in the scater (McCarthy et al., 2017) package, which in turn relies on uwot (https://www.github.com/jlmelville/uwot). Features were scaled to unit variance and the 15 nearest neighbors were considered for embedding. UMAP coordinates were then exported for visualization using ggplot2. FlowSOM (Van Gassen et al., 2015) uses self-organizing maps (SOM) to efficiently categorize cytometry data into non-overlapping cell populations and was performed using CATALYST (default parameters, target: 100 clusters, 50 metaclusters). After manual inspection, we merged two phenotypically similar clusters to avoid overclustering (Saeys et al., 2017) and ended up with 49 discrete T cell clusters. The R/Bioconductor package ComplexHeatmap was used to visualize T cell phenotypes and the arcsine transformed signal intensity of each marker was independently scaled using a 0–1 transformation across all clusters.

To analyze differential cluster abundance, we used the workflow laid out by Nowicka et al. (2017). FlowSOM cluster cell counts were modeled linearly with time point as a dependent categorical variable and volunteer as a fixed effect using the diffcyt (Weber et al., 2019) implementation of edgeR (Robinson et al., 2010). The edgeR functions automatically normalize cluster counts for the total number of cells and improve statistical power by sharing information on cluster count variance between clusters. Pairwise comparisons were performed relative to baseline, and clusters with an FDR < 0.05 and absolute fold-change >2 were deemed to vary significantly through time. We assessed differential cluster abundance independently for volunteers receiving their first or third infection. We also assessed whether marker expression varied significantly through time, and to do this, we merged clusters belonging to the same T cell lineage according to their expression of CD4, CD8, CD56, Vδ2, and Vα7.2. Adaptive CD4^+^ and CD8^+^ T cells were then split into naive, effector, effector memory, and central memory subsets based on their expression of the markers CD45RA, CD45RO, CD57, and CCR7. All regulatory T cells (CD4^pos^ CD25^hi^ CD127^neg^) were merged into a single cluster. Linear models derived from the limma package, which is optimized for continuous data, were then used to independently assess differential marker expression relative to baseline using pairwise comparisons with moderated *t* tests; a shift in median expression of at least 10% and an FDR < 0.05 were required for significance. Results were visualized using ComplexHeatmap with row-wise z-score transformed marker intensities shown for each lineage (or subset) of T cells.

### Meta-analysis of liver injury

A surrogate dataset from Reuling et al. (2018) was used to assess the risk of liver injury during a first-in-life infection compared with rechallenge. We extracted data from every CHMI study that used the 3D7 *P. falciparum* clone (or its parental NF54 line), initiated infection via mosquito bite or direct blood challenge, and that had a treatment threshold based on thick smear positivity (estimated to be at least 5,000 parasites ml^−1^ blood). This produced data for 95 volunteers across seven CHMI studies. Notably, Reuling et al. used longitudinal data to show that LFT abnormalities peaked up to 6 days after treatment in line with our own T6 time point. Furthermore, in every CHMI study (including our own), liver function test (LFT) abnormalities were graded using the same adaptation of the WHO adverse event grading system. An LFT reading >1.0 but <2.5 times the upper limit of normal was graded as mild; a reading >2.5 but <5.0 times the upper limit was graded moderate; and a reading >5.0 times the upper limit was graded severe. For ALT, the upper limit of normal was 35 or 45 U liter^−1^ for female and male volunteers, respectively. Data from the 95 volunteers in Reuling et al. and the 3 volunteers undergoing first infection in our VAC063C study were pooled for analysis, and we calculated a weighted peak parasitemia across the cohort by using the mean number of parasites ml^−1^ and the number of volunteers in each of the eight CHMI studies. To statistically test whether an abnormal ALT reading was more prevalent in the 98 volunteers experiencing their first malaria episode compared with the eight volunteers undergoing rechallenge (either second or third infection), we used Barnard’s test, which examines the association of two independent categorical variables in a 2 × 2 contingency table. A P value below 0.05 was considered significant.

### Pearson correlation analysis

To assess the relationship between liver injury, T cell activation, systemic inflammation, and the clinical symptoms of malaria, we constructed a Pearson correlation matrix using our VAC063C dataset. Importantly, this analysis was agnostic of infection number (all volunteers were included). We input the concentration of ALT and the percentage of activated effector (effector memory) CD4^+^ T cells, regulatory T cells, and cytotoxic (granzyme B^pos^) T cells (all at T6); the fold-change of lymphocytes, hemoglobin, and all significant plasma analytes (those shown in Fig. S2 A), which were calculated at diagnosis or T6 (relative to baseline) according to their largest absolute fold-change; the maximum parasite density and maximum core temperature (at any time point up to 48 h after treatment); and the titers of AMA1/MSP1-specific class-switched antibodies (measured 28 days after challenge). All data were log_2 _transformed and Pearson correlation was performed in R using the corrplot function. Correlation coefficients were used for unsupervised hierarchical clustering by Euclidean distance.

### In vitro cytotoxicity

The hepatoma cell line HepG2 was kindly provided by Shaden Melhem (University of Edinburgh, Edinburgh, UK). HepG2 cells were maintained at 37°C and 5% CO_2_ in RPMI 1640 supplemented with 10% heat-inactivated FBS (FBS Premium Plus, #16000044; Gibco), 1 × GlutaMAX (#35050061; Gibco), 100 U ml^−1^ penicillin, and 100 μg ml^−1^ streptomycin (#15140122; Gibco). The expansion was performed in tissue culture–treated culture flasks (#430641U; Corning) and cells were detached using TrypLE Express Enzyme (#12604013; Gibco). For cytotoxicity assays, HepG2 cells were seeded at a density of 5.5 × 10^5^ cells per well in 96 well tissue culture–treated plates precoated with poly-D-lysine (#A3890401; Gibco) and incubated for 24 h to allow the formation of a confluent monolayer.

The next day PBMC were thawed, washed, counted, and resuspended at a concentration of 3 × 10^6^ cells ml^−1^ in RPMI 1640 supplemented with 10% FBS (FBS Premium Plus), 2 mM L-Glutamine (#25030081), 1 mM sodium pyruvate (#11360070), 10 mM HEPES (#15630080), 500 µM β-mercaptoethanol (#31350010), and 1x MEM non-essential amino acids (#M7145) (all Gibco except amino acids from Sigma-Aldrich). Cells were transferred to ultra-low attachment plates (#3473; Corning) and stimulated with cell activation cocktail (#423302; BioLegend) for 3 h at 37°C and 5% CO_2_, the working concentration of PMA and ionomycin used was 40.5 and 669.3 nM, respectively. PBMC were then harvested, washed two times, and added onto HepG2 monolayers at increasing ratios of effector (PBMC) to target cells (HepG2). After 24 h of coculture, the release of lactate dehydrogenase (LDH) from damaged HepG2 cells was measured in the culture supernatant using the CyQUANT LDH cytotoxicity assay according to the manufacturer’s instructions (#C20301; Invitrogen). Background LDH release was quantified in wells containing only HepG2 cells. Absorbance was read on a FLUOstar Omega plate reader.

### Online supplemental material

Fig. S1 presents the results of maximum likelihood estimation to select the best model fit for the frequency of severe or complicated malaria across the first 14 infections of life (relates to Fig. 1). It also displays the signature genes upregulated in peripheral blood in response to *P. falciparum* (relates to Fig. 2). Fig. S2 highlights the plasma analytes and T cell genes upregulated in response to *P. falciparum* (relates to Figs. 3 and 4, respectively). Fig. S3 outlines the experimental plan for single-cell RNAseq analysis and a working model for the bifurcation of activated CD4^+^ T cells along the TCF1-BLIMP1 axis (relates to Fig. 5). Finally, Figs. S4 and S5 show changes in cluster frequency and protein marker expression on T cells during infection and convalescence (both relate to Fig. 6). Table S1 provides demographic information for each volunteer infected and rechallenged with *P*.* falciparium*. Table S2 lists the signature genes associated with the terminal differentiation or self-renewal of CD4^+^ T cells. Table S3 provides details on the antibodies used for flow-sorting and mass cytometry. Table S4 provides the correlation coefficients for the matrix shown in Fig. 7.

## Supplementary Material

Table S1 shows demographics of volunteers infected and rechallenged with *P*.* falciparium* (3D7) during VAC063A-C and includes genetic and non-genetic variables known to influence human immune variation in vitro.

Table S2 shows signature genes associated with terminal differentiation and self-renewal in CD4^+^ T cells.

Table S3 shows mass cytometry antibody panel for T cell fate and function in VAC063C and includes information on antibody clone, source, and heavy metal conjugate. Also shown (on tab two) is the antibody panel used for the flow-sorting of CD4^+^ T cell subsets.

Table S4 shows coefficient and P values underlying the Pearson correlation matrix shown in Fig. 7.

## Data Availability

All bulk RNAseq data (whole blood and sorted T cell subsets) have been deposited in NCBI’s Gene Expression Omnibus and are accessible through GEO SuperSeries accession number GSE172481. Single-cell RNAseq data have also been deposited in NCBI’s Gene Expression Omnibus and are available through accession number GSE275092. Bulk TCRβ sequencing data have been deposited in the European Nucleotide Archive and are available through accession number PRJEB71976. CyTOF (mass cytometry) data have been deposited at https://flowrepository.org, and these can be accessed through experiment numbers FR-FCM-Z47Z (VAC063C), FR-FCM-Z3HA (VAC069A), and FR-FCM-Z465 (VAC069B). All other data are available in the figures, supplementary figures, and supplementary tables.
